# ADSCs Promote Tenocyte Proliferation by Reducing the Methylation Level of lncRNA Morf4l1 in Tendon Injury

**DOI:** 10.3389/fchem.2022.908312

**Published:** 2022-07-04

**Authors:** Haibo Zhao, Wei Chen, Jinli Chen, Chao Qi, Tianrui Wang, Jing Zhang, Di Qu, Tengbo Yu, Yingze Zhang

**Affiliations:** ^1^ Department of Orthopedics, Affiliated Hospital of Qingdao University, Qingdao, China; ^2^ Third Affiliated Hospital of Hebei Medical University, Shi Jiazhuang, China

**Keywords:** adipose-derived mesenchymal stem cells (ADSCs), tendon injury, tendon repair, DNA methylation, lncRNA

## Abstract

**Objective:** Tendons are the special connective tissue that connects bones to muscles and governs joint movement in response to loads passed by muscles. The healing of tendon injuries is still a challenge. In recent years, adipose-derived mesenchymal stem cells (ADSCs) have been increasingly used for tissue regeneration, but the underlying mechanism of tendon injury still remains unclear.

**Methods:** High-throughput sequencing was used to identify a novel lncRNA, whose expression was significantly decreased in injured tendon compared with normal tendon. Furthermore, pyrosequencing, nuclear-cytoplasmic separation, FISH assay and qRT-PCR analysis were used to verify the level of lncRNA methylation in the injured tenocytes. lncRNA was confirmed to promote the proliferation of tenocytes by flow cytometry, wound healing assay, qRT-PCR, and western blot, and the target gene of lncRNA was predicted and verified. To confirm that ADSCs could repair injured tendons, ADSCs and injured tenocytes were co-cultured *in vitro*, and ADSCs were injected into injured tendons *in vitro*, respectively.

**Results:** The lncRNA Morf4l1 promoter methylation in injured tendons led to down-regulation of its expression and inhibition of tenocyte proliferation. LncRNA Morf4l1 promoted the expression of TGF-β2 by targeting 3′U of miR-145-5p. After co-cultured ADSCs and injured tenocytes, the expression of lncRNA Morf4l1 was up-regulated, and the proliferation of injured tenocytes *in vitro* was promoted. The ADSCs were injected into the injured tendon to repair the injured tendon *in vivo*.

**Conclusion:** This study confirmed that ADSCs promoted tendon wound healing by reducing the methylation level of lncRNA Morf4l1.

## Summary Box

### What Are the New Findings?

1) We found a novel lncRNA that has not been reported in tendon repair.
2) LncRNA Morf4l1 promoter produced methylation in injured tendons, promoted the proliferation of injured tenocytes after methylation inhibition, and reduced oxidative stress in injured tenocytes.
3) LncRNA Morf4l1 promoted the expression of TGF-β2 by targeting 3′U of miR-145-5p.
4) ADSCs increased the expression of lncRNA Morf4l in injured tenocytes to achieve the purpose of repairing tendon injury.

## Background

Tendons are the special connective tissue that connects bones to muscles and governs joint movement in response to loads passed by muscles (Andarawis-Puri et al., 2015). Tendon injuries can be classified as either chronic (e.g., tendonitis and tendinopathy) or acute (e.g., complete tearing of the tendon due to high tensile loads) (Järvinen et al., 2005; Sharma and Maffulli, 2008). Mesenchymal stem cells (MSCs) therapy is more effective and safer than traditional methods, such as physical therapies, non-steroid anti-inflammation drugs (NSAIDs) intake, and administration of steroid injections (Dakin et al., 2015; Liu et al., 2017; Zhang et al., 2018; Han et al., 2019; Gissi et al., 2020), in the treatment of tendinopathy. These approaches are limited to pain relief. There are many sources of mesenchymal stem cells, common ones include bone marrow derived mesenchymal stem cells (BM-MSCs), umbilical cord derived mesenchymal stem cells (ADSCs) and so on (Ben-David and Benvenisty, 2011). Although BM-MSCs is recognized immune compatible, effective, safe, suitable for various regenerative studies and relatively inexpensive to treat, its major disadvantages are the small number of cells produced during invasive harvesting and the pain they cause to patients (Jaiswal et al., 2000). ADSCs also have many advantages in clinical applications because its relatively easy to obtain in humans or other experimental bodies (Nakagami et al., 2006). ADSCs are gradually replacing BM-MSCs due to their advantages of convenient sampling and abundant sources. However, the exact molecular mechanisms of ADSCs therapy still remain unclarifed.Therefore, identifying specifc biomarkers to develop effective targeted therapy strategies is of urgency for the improvement in the repair of tendon injuries.

In recent years, DNA methylation has attracted more and more attention from epigenetics and pathologic prevention and treatment. DNA methylation products are called 5-methylcytosine (5-MC) and usually occur in CpG islands (Deng et al., 2018). CpG islands are mainly CpG dinucleotide rich regions, especially promoter methylation plays an important role in gene regulation (Illingworth et al., 2010; Bellacosa and Drohat, 2015) The GC content of CpG islands is greater than 50% (Robertson and A.Jones, 2000; Wong and Chim, 2015). DNA methylation is involved in many biological activities, such as gene transcription, gene structure stabilization, cell differentiation and foreign body metabolism. Methylation often plays a role in gene silencing in gene regulated transcription. In nasopharyngeal carcinoma, for example, HOPX has high methylation levels in cancerous tissues, and patients with HOPX hypermethylation have a poor prognosis (Ren et al., 2017). In liver cancer, hypermethylation in the pre-5kb gene region of miR-122 promoter in cancer tissues results in decreased expression of miR-122 and promotes cancer cell viability. (Cheng et al., 2018). However, the role of DNA methylation of lncRNA in tendon repair is rarely reported.

In this study, we analyzed lncRNA high-throughput data to identify potential lncRNA Morf4l1, specifically low-expressed in injured tendons and determined the promoter methylation level of lncRNA Morf4l1. lncRNA Morf4l1 promoted tendon repair after demethylation and ADSCs treatment promoted tendon repair after lncRNAMorf4l1 methylation *in vivo* and *in vitro*. Our data provided evidence that DNA methylation of lncRNA Morf4l1 promoter was involved in tendon injury and ADSCs rescued tendon injury, which might provide a novel prognostic biomarker and therapeutic target for tendon repair.

## Materials and Methods

### Animal Models and Experimental Design

The healthy male Sprague-Dawley (SD) rats (Ten-week-old; Guangzhou Medical Laboratory Animal Center; China) were randomly divided into three groups (*n* = 3 per group). One group didn’t receive any intervention (as control group), while tendons of rats in the other two groups were cut for a week. One of the two groups was injected with ADSCs (1×10^6^ cells per rat) into the injured tendons (as injury + ADSCs group), followed by continuous injection once a day for 4 weeks; the other group was injected with the same volume of saline once a day for 4 weeks (as injury group). Routine injection of penicillin was administered to prevent infection after operation. All rats were euthanized after four weeks. This study was approved by the Ethics Committee of Qingdao University Hospital (Shandong, China).

### Isolation and Identification of ADSCs

Simply put, male SD rats were anesthetized by intraperitoneal injection of pentobarbital sodium. After disinfection, the skin of the rats was cut open and adipose tissue was removed. Adipose tissue was shredded and digested with 0.1% type I collagenase and 0.05% trypsin (Gibco, Grand Island, NY, United States). Suspended tissues were cultured in low-glucose Dulbecco’s modified Eagle’s medium (DMEM; Gibco). The medium was supplemented with 10% (V/V) fetal bovine serum (FBS) and 100 U/mL penicillin-streptomycin (Gibco). The third generation of ADSCs was used and phenotypic analysis was performed. For detailed methods of isolation, purification and identification of ADSCs, refer to Xie M, 2017 (Xie et al., 2017).

### Cell Culture

The tenocytes and 293T cells (Fenghbio, China) were cultured in DMEM. Rat adipose tissue-derived mesenchymal stem cells (Percell, China) were cultured in the complete medium (Cat. CM-R198). All tenocytes were treated with serum starvation for 12 h. Normally cultured tenocytes served as a control group. In the injury group, 200 μmol/L H_2_O_2_ (Solarbio, China) was added to the medium when tenocytes reached 70–80% confluence in 60 mm culture dishes. The drug was replaced every 24 h for 72 h. Similarly, in the injury + demethylation group, 10 μmol/L DNA methylase inhibitor 5-Aza-CdR (Sigma-Aldrich, United States) was supplemented on the basis of injury group. All cells were cultured in an atmosphere at 37°C with 5% CO_2_.

### Cell Co-cultured

ADSCs and tenocytes were co-cultured by the Transwell chamber (0.4 μm, 6-well plates, Corning, United States). ADSCs were loaded in the upper chamber and cultured in the complete medium. The tenocytes were seeded in the 6-well plates and cultured in the DMEM supplemented with 10% FBS, while 200 μmol/L H_2_O_2_ was added to the medium. ADSCs and tenocytes were co-cultured for 48 h and then proceeded to follow-up experiments.

### Cell Transfection

pcDNA 3.1/lncRNA Morf4l1, si-lncRNA Morf4l1, miR-145-5p mimics and miR-145-5p inhibitor were obtained from RiboBio (China). Empty vector (pcDNA 3.1), si-NC, mimics NC and inhibitor NC were used as a negative control (NC). The transfected plasmid was transfected when the cells reached about 70–80% fusion in a 60 mm dish, and the cells were collected 48 h later.

### Enzyme-Linked Immunosorbent Assay (ELISA)

The levels of LDH (Yanjin, China), CK (Xitang, China), SOD (Chuangxiang, China) and MDA (Duma, China) in tendon tissue were measured using ELISA kits. The assay was conducted following the manufacturer’s protocols. Each well was detected for the OD value at 450 nm by Perlong DNM-9602 Microplate reader (China).

### Wound Healing Assay

The tenocytes grown to near-confluence in 6-well plates were subjected to serum-free medium for 24 h of starvation (0.8 μm, Corning, United States). The monolayers were scratched using a sterile 10 μL pipette tip. Photographs of tendinocyte migration at the corresponding wound site were observed at 0 and 24 h (IX73, Olympus, Japan).

### CCK-8 Cell Proliferation Assay

According to the manufacturer’s instructions (Dojindo Laboratories, Japan), 1×10^3^ cells were seeded in 96-well plates and cultured for 24 h. The cell proliferation assay was performed at 12, 24, 48, and 72 h. Add 10 μL of CCK-8 reagent to each well and incubate at 37°C for 2 h. Record the absorbance at 450 nm (Tecan Group Ltd., Switzerland).

### Flow Cytometry Analysis

Cell apoptosis was detected by harvesting 4×10^5^ tenocytes and staining using an Annexin V-FITC/PI Apoptosis Detection kit (Liankebio, China). Cell oxidative stress was detected by harvesting 1×10^7^ tenocytes and staining using a Reactive oxygen species assay kit (Solarbio, China). Cells were analyzed by flow cytometry using a BD FACS CantoII instrument within 1 h. Refer to the flow cytometry kit instructions for detailed procedures. Cells were analyzed by flow cytometry using a BD FACS CantoII instrument within 1 h.

### TUNEL Assay

The apoptosis level of tendon tissue was detected by using TUNEL apoptosis assay kit-FITC according to the manufacturer’s instructions (Boster, China). Frozen sections were fixed with 4% paraformaldehyde for 1 h, and then digested by proteinase K for 10 min. Subsequently, frozen sections were incubated successively with TdT and BIO-d-UTP. Finally, blocking solution and SABC were added to connect the fluorescent radical group. Images were captured using a laser confocal microscope (Olympus, Japan).

### Luciferase Reporter Assays

Luciferase reporter plasmids that contained the wild-type and mutant of the lncRNA Morf4l1 and TGF-β promoter were constructed. The wild-type (pmirGLO-lncRNA Morf4l1 3′UTR wt or pmirGLO-TGF-β 3′UTR wt) and mutant (pmirGLO-lncRNA Morf4l1 3′UTR mut or pmirGLO-TGF-β 3′UTR mut) of luciferase reporter plasmids were co-transfected with miR-145-5p mimic or NC into 293T cells for 48 h. Luciferase activity was measured by Promega (United States).

### Nuclear-Cytoplasmic Separation

Nuclear and cytoplasmic fractions were isolated from tenocytes using a PARIS kit (Ambion, United States). Then, 1×10^7^ tenocytes were collected and suspended in cell partial buffer and incubated with ice for 10min. After centrifugation, After centrifugation, RNA was extracted from the preserved supernatant and nuclear particles. GAPDH and U6 served as cytoplasmic and nuclear controls, respectively. qRT-PCR was used to detect the expression levels of lncRNAMorf4l1, GAPDH and U6 in cytoplasmic and nuclear components.

### RNA Fluorescence *in situ* Hybridization (FISH) Assay

Tenocytes were cultured in 6-chamber slides for 24 h, fixed with paraformaldehyde (4%) for 20 min. It is then dehydrated in an ethanol solution. The cells were hybridized with the probe 42°C overnight (Servicebio, China). After the non-specific probe was removed, the second labeled probe was used to detect the biotin-labeled lncRNA Morf4l1. Finally, the nuclei were stained with DAPI (Solarbio, China) and the images were obtained using a confocal microscope (Olympus, Japan).

The probe of lncRNA Morf4l1 used was as follows:

CCT​GGA​ATT​TAG​GCT​TCG​GGT​CCT​GCT​TGG (ttt CAT​CAT​CAT​ACA​TCA​TCA​T)_30+-_.

The second labeled probe of lncRNA Morf4l1 used was as follows:

tt ATGATGATGT ATGATGATGT.

### Immunofluorescence Staining

Simply put, tenocytes were fixed with paraformaldehyde for 15 min. Following blocking with 5% FBS for 1 h, the cells were incubated with primary antibodies against TGF-β2 (Proteintech, United States, 1:200) at 4°C overnight. Then, the tenocytes were stained with secondary antibodies at 37°C for 1 h (Abcam, United States, 1:500). DAPI was used to stain the nucleus. Images were captured using a laser confocal microscope (Olympus, Japan).

### Quantitative Real-Time Polymerase Chain Reaction (qRT-PCR)

Total RNA was extracted from tenocytes using Trizol reagent (TakaRa, Japan). A total of 1 μg of RNA was reverse-transcribed using the PrimeScript"RT reagent Kit with gDNA Eraser (TakaRa, Japan), followed by qRT-PCR with the kitspecification of SYBR@ Premix Ex Taqm II (TakaRa, Japan) on iQ5 Real-Time PCR System (BioRad, United States). The results were normalized using GAPDH for lncRNA Morf4l1 or U6 for miR-145-5p and analysed according to 2^−ΔΔCT^ method. The primers (RiboBio, China) were listed in Table 1.

**TABLE 1:** Primer sequence.

Gene	Forward (5′–3′)	Reverse (5′–3′)
GADPH	ATC​TCG​CTC​CTG​GAA​GAT​GG	CAA​GTT​CAA​CGG​CAC​AGT​CA
U6	CTCGCTTCGGCAGCACA	AAC​GCT​TCA​CGA​ATT​TGC​GT
lncRNA Morf4l1	GTG​TAT​GGA​GCG​CCA​CAC​TTA	GGC​ACT​GAA​CAG​AGT​TGC​AGA
miR-145-5p	ATTTCGCTGCTCCATTTA	ATTTCGCTGCTCCATTTA
TGF-β2	ATG​TGC​AGG​ATA​ATT​GCT​GCC	TGG​TGT​TGT​ACA​GGC​TGA​GC

### Western Blot

The total protein separated by SDS-PAGE were transferred onto polyvinylidene difluoride (PVDF) membranes. Then the PVDF membrane was blocked with non-fat milk powder (1 h at room temperature), and the primary antibody (4°C for overnight) and secondary antibody (37°C for 1 h) were incubated. The primary and secondary antibodies were shown in Table 2. The gray value of each band was detected by chemiluminescence substrate (Boster, China).

**TABLE 2:** Antibody information.

Antibody name	Antibody manufacturer	Manufacturer country	Antibody concentration
PCNA	Abcam	United Kingdom	1:1000
Caspase3	Abcam	United Kingdom	1:1000
Bcl-2	Abcam	United Kingdom	1:1000
Bax	Abcam	United Kingdom	1:1000
TGF-β2	Abcam	United Kingdom	1:1000
GAPDH	Abcam	United Kingdom	1:8000

### HE Staining

Frozen sections were fixed with pre-cooled acetone for 10–15 min at room temperature. After 5 min staining with hematoxylin, it was differentiated with 1% hydrochloric acid alcohol. Then, sections were dehydrated with alcohol, transparentized by xylene, and sealed with gum. Images were acquired on an Olympus BH2 microscope (Olympus, Japan).

### Statistical Analysis

All experiments were performed in triplicate. Results are presented as the means ± SD. Data were analyzed using unpaired Student’s t-test (two-tailed, with *p* < 0.05 considered significant). The GraphPad Prism 5 (GraphPad Software Inc., La Jolla, United States) was used for data analysis.

## Results

### Hypermethylation of lncRNA Morf4l1 in Injured Tendons Tissues

**FIGURE 1:**
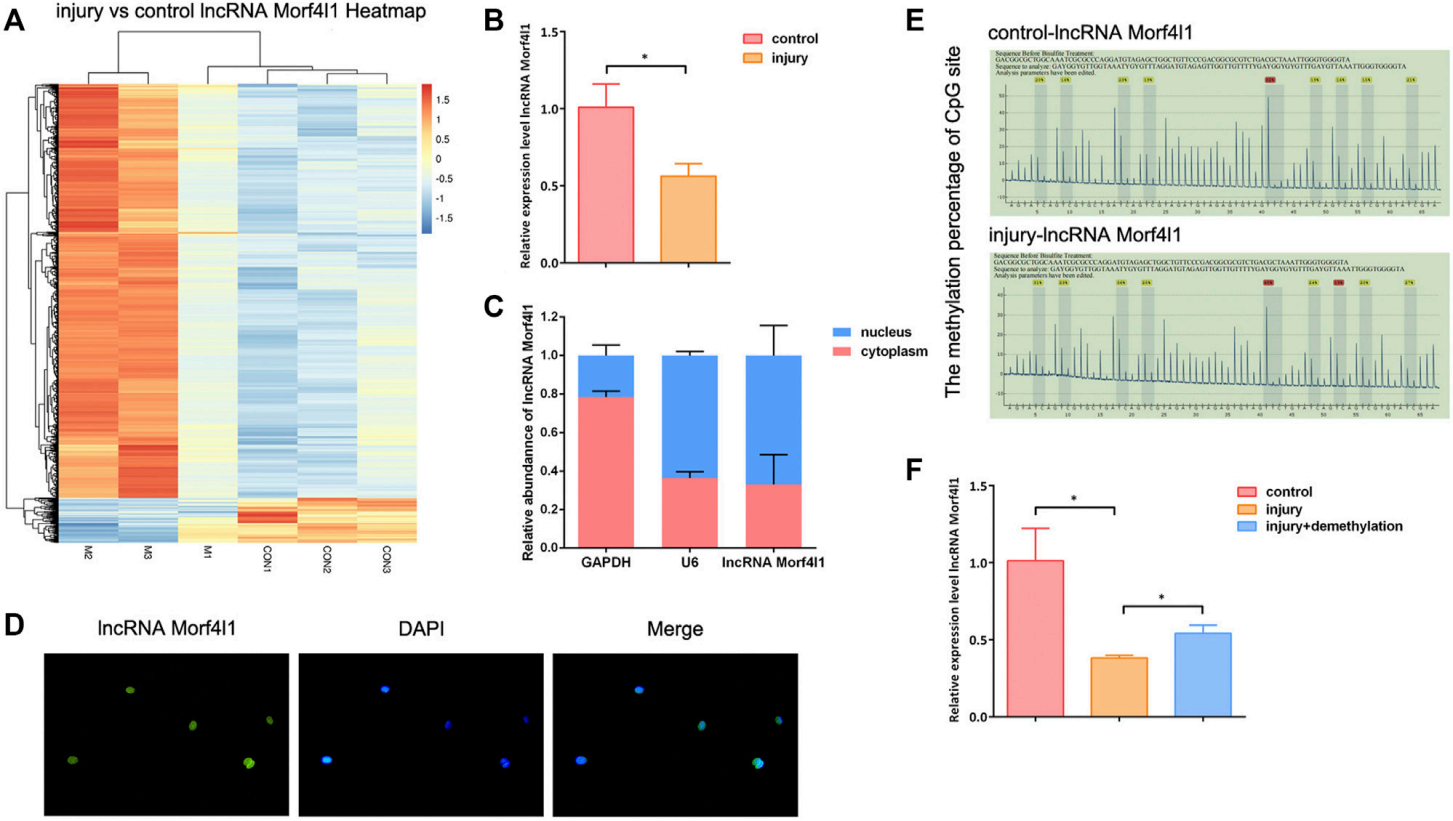
LncRNA Morf4l1 was hypermethylated in injured tendon tissues. **(A)** Heatmap clustering of the differentially expressed lncRNA in normal tendon tissues and injured tendon tissues. Columns: individual samples; rows: lncRNA; blue: low expression; orange: high expression **(B)** qRT-PCR assay was performed to detect the expression of lncRNA Morf4l1 in normal tendon tissues and injured tendon tissues **(C,D)** Nucleus-cytoplasm separation and RNA-FISH assay were used to determine lncRNAMorf4l1 subcellular localization. DAPI was used to stain nuclei (blue); Left: green fluorescence was from the biotin fusions; Right: the merged image; Base magnification:×400 **(E)** Pyrosequencing report showed that the methylation level of the lncRNAMorf4l1 promoter region was higher in injured tendons compared with normal tendons. Gray: the actual detection of the fluorescence signal intensity of the methylation site C/T; Yellow: the internal reference control, which was used to monitor the conversion efficiency of sulfite; Each peak is the fluorescence intensity of the sequence to be tested, and the top percentage is the C/T fluorescence signal ratio, that is, the methylation level of the methylation site to be detected **(F)** qRT-PCR assay was used to detect the expression of lncRNA Morf4l1 after demethylation of injured tenocytes (*n* = 3; Mean ± SD, ^*^ *p* < 0.05, ^**^ *p* < 0.01, ^***^ *p* < 0.001; Student’s t-tests).

Potential specifically expressed lncRNA in injured tendon tissues were identify by high-throughput sequencing. The results showed that lncRNA Morf4l1 expression was significantly down-regulated in injured tendons compared with normal control (Figure 1A), which was verified by qRT-PCR assay (Figure 1B). To further explore the underlying mechanism of lncRNA Morf4l1 affecting tendon repair, subcellular localization of lncRNA Morf4l1. Subcellular localization assay and RNA-FISH assay showed that lncRNAMorf4l1 was mainly distributed in the nucleus (Figures 1C,D). To confirm whether the promoter of lncRNA Morf4l1 was modified by methylation, pyrosequencing analysis was performed to examine the methylation levels in injured tendons and normal tendons. As shown in Figure 1E, lncRNA Morf4l1 promoter region CpG island detected nine methylation sites. And each site was remarkably hypermethylated compared with the control group. Subsequently, demethylation drugs 5-Aza-CdR was used to treat injured tenocytes, and qRT-PCR assay results showed that lncRNA Morf4l1 expression was down-regulated after treatment (Figure 1F). These results suggested that lncRNA Morf4l1 was modified by methylation and its expression was down-regulated in injured tendons.

### LncRNA Morf4l1 Inhibited Oxidative Stress and Promoted Proliferation of Tenocytes

**FIGURE 2:**
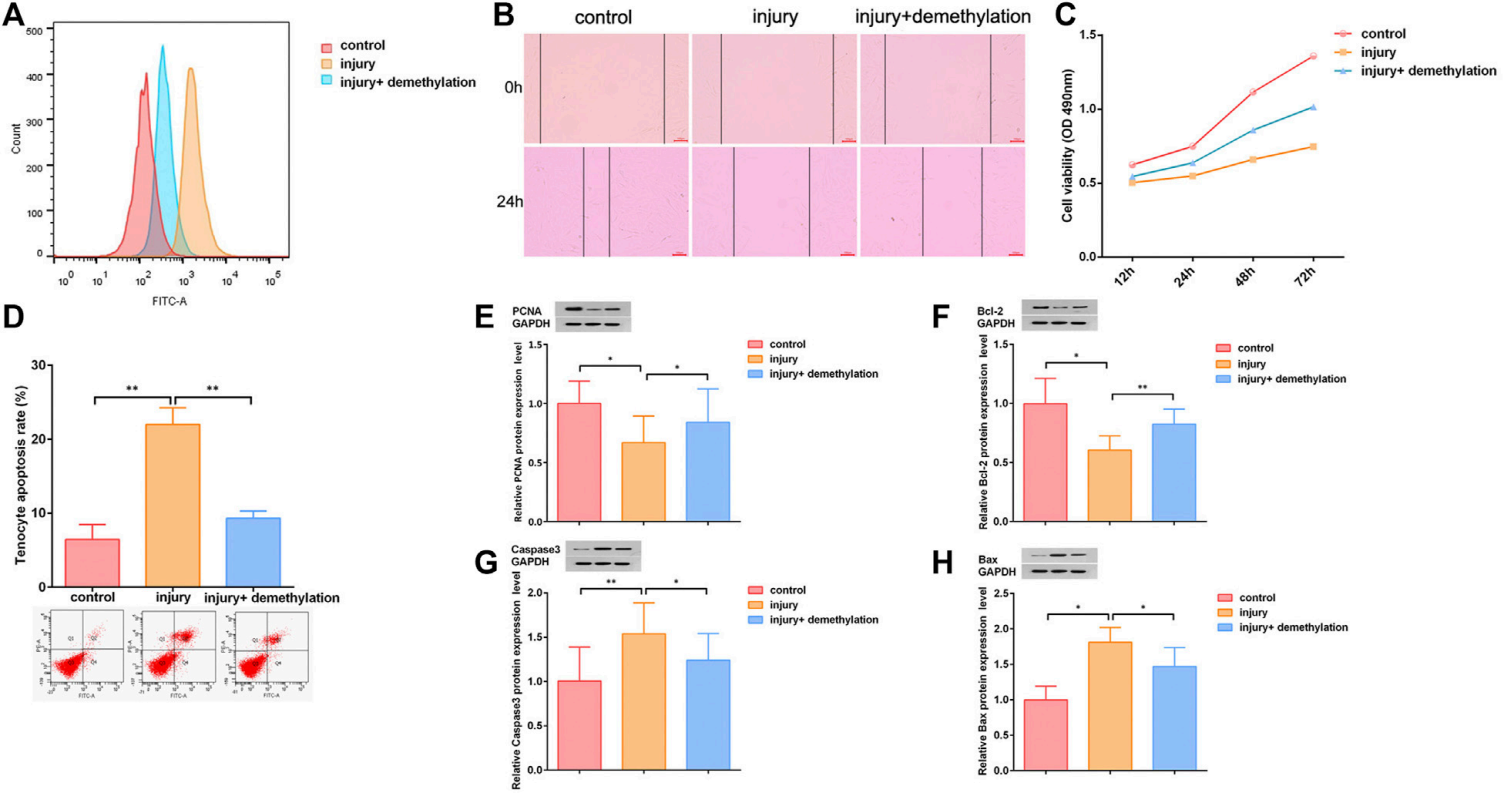
LncRNA Morf4l1 inhibited oxidative stress and promoted proliferation of tenocytes. **(A)** Flow cytometry analysis was used to detect the level of ROS. The oxidative stress level of injured tenocytes was reduced after demethylation **(B)** Wound-healing was used to detect the effect of demethylation on the migration ability of injured tenocytes; Scale: 100 μm **(C)** CCK-8 assay was used to detect the proliferation ability of injured tenocytes after demethylation **(D)** Flow cytometry analysis was used to detect the apoptosis of injured tenocytes after demethylation **(E–H)** Western blot was performed to detect the expression of PCNA, Bcl-2, Caspase3 and Bax of injured tenocytes after demethylation (*n* = 3; Mean ± SD, *p < 0.05, **p < 0.01, ***p < 0.001; Student’s t-tests).

We next detected the effect of lncRNA Morf4l1 on the oxidative stress of tenocytes. Flow cytometry analysis results showed that lncRNA Morf4l1 methylation promoted oxidative stress in injured tenocytes (Figure 2A). To determine whether lncRNA Morf4l1 regulated the migration and proliferation of tenocytes, wound healing assay, CCK-8 assay, flow cytometry and western blot assay were conducted. The methylation of lncRNA Morf4l1 significantly suppressed tenocyte migration (Figure 2B). CCK-8 assay displayed that methylation of lncRNA Morf4l1 significantly inhibited tenocyte proliferation, as in demethylation of lncRNA Morf4l1 (Figure 2C), which were also verified in apoptosis assay by flow cytometry (Figure 2D). PCNA and Bcl-2 are cell proliferation-related proteins, and Caspase3 and Bax are cell apoptosis-related proteins. Western blot assay confirmed that methylation of lncRNA Morf4l1 significantly inhibited the expression of PCNA and Bcl-2, and promoted the expression of Caspase3 and Bax, which was consistent with the tendency in demethylation of lncRNA Morf4l1 (Figures 2E–H). These findings illustrated that lncRNA Morf4l1 inhibited oxidative stress and promoted proliferation of tenocytes.

### LncRNA Morf4l1 Promoted the Expression of TGF-β2 Through Targeting 3′U of miR-145-5p

**FIGURE 3:**
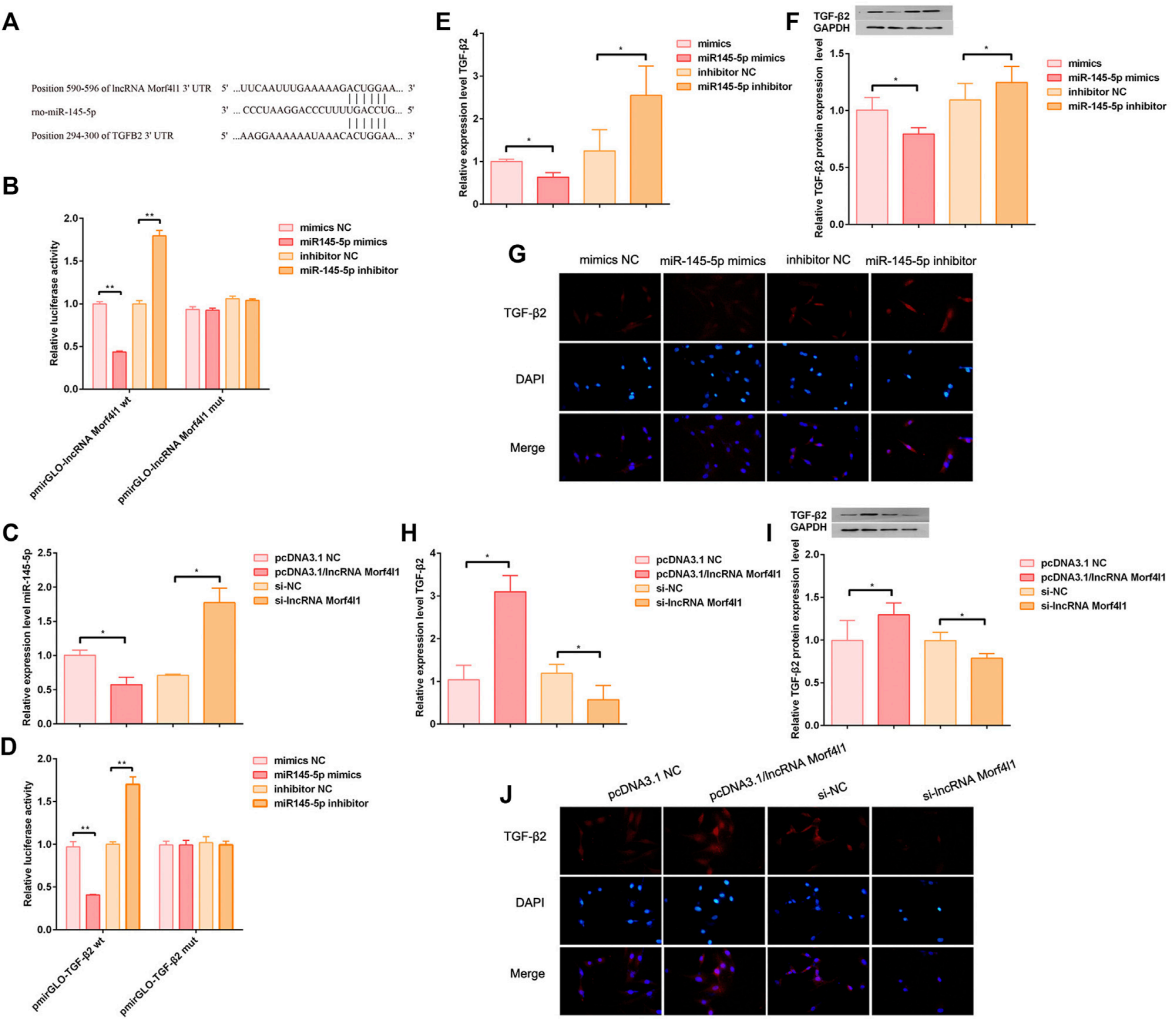
LncRNA Morf4l1 promoted the expression of TGF-β2 through targeting 3′U of miR-145-5p. **(A)** Targetscan was performed to predict putative binding sites **(B)** Luciferase reporter analysis was used to detect the bindings between lncRNA Morf4l1 and miR-145-5p in 293T cell. Overexpression or inhibition of miR-145-5p in the lncRNA Morf4l1 wild type (wt) enhanced or weakened the luciferase activity, while the lncRNA Morf4l1 mutant type (mut) did not change **(C)** The effect of lncRNA Morf4l1 intervention on the expression of miR-145-5p in tenocytes was evaluated using qRT-PCR **(D)** Luciferase reporter analysis was used to detect the bindings between miR-145-5p and TGF-β2 in 293T cell. Overexpression or inhibition of miR-145-5p in the TGF-β2 wild type (wt) enhanced or weakened the luciferase activity, while the TGF-β2 mutant type (mut) did not change **(E)** The effect of miR-145-5p intervention on the expression of TGF-β2 in tenocytes was evaluated using qRT-PCR **(F)** The effect of miR-145-5p intervention on the expression of TGF-β2 in tenocytes was evaluated using Western blot **(G)** The results of immunofluorescence assay showed that miR-145-5p was overexpressed or inhibited, affecting the expression of TGF-β2. DAPI was performed to stain nuclei (blue); the above red fluorescence intensity was positively correlated with the expression of TGF-β2; Under the merged image; Base magnification:×200 **(H)** The effect of lncRNA Morf4l1 intervention on the expression of TGF-β2 in tenocytes was evaluated using qRT-PCR **(I)** The effect of lncRNA Morf4l1 intervention on the expression of TGF-β2 in tenocytes was evaluated using Western blot **(J)** Immunofluorescence assay results showed that lncRNA Morf4l1 was overexpressed, thus promoting the expression of TGF-β2, and vice versa; Base magnification:×200 (*n* = 3; Mean ± SD, *p < 0.05, **p < 0.01, ***p < 0.001; Student’s t-tests).

Targetscan was used to predict that miR145-5p 3′UTR had putative binding sites of lncRNA Morf4l1, and TGF-β2 3′UTR had putative binding sites of miR145-5p (Figure 3A). To verify this prediction, the luciferase reports was assessed the luciferase activities of the wild type or mutant promoter reporter gene of lncRNA Morf4l1 in 293T cells. The overexpression of miR145-5p markedly decreased the luciferase activities, while knockdown of miR145-5p significantly increased the luciferase activities; the activities of the mutant reporter gene were not affected (Figure 3B). qRT-PCR demonstrated that overexpression of lncRNA Morf4l1 significantly decreased the expression of miR145-5p in tenocytes, which was in accordance with the tendency in suppression of lncRNA Morf4l1 (Figure 3C). Similarly, the luciferase report was used to evaluate the luciferase activities after miR145-5p and TGF co-transfection (Figure 3D), and the qRT-PCR was used to evaluate the expression level of TGF-β2 after miR145-5p was inhibited or overexpressed (Figure 3E), which were consistent with the tendency in Figures 3B,C. Immunofluorescence assay (Figure 3G) showed that overexpression of miR145-5p significantly reduced the level of protein expression, as compared with significantly increased expression after inhibition of miR145-5p (Figure 3F).

Similarly, TGF-β2 mRNA and protein expression levels were detected after lncRNAMorf4l1 was inhibited or overexpressed. These results showed lncRNA Morf4l1 promoted the expression of TGF-β2 (Figures 3H–J). Taken together, these findings demonstrated that lncRNA Morf4l1 promoted the expression of TGF-β2 by regulating the miR145-5p/TGF-β2 axis.

### Co-Cultured Tenocytes and ADSCs to Rescue the Tendon Injury

**FIGURE 4:**
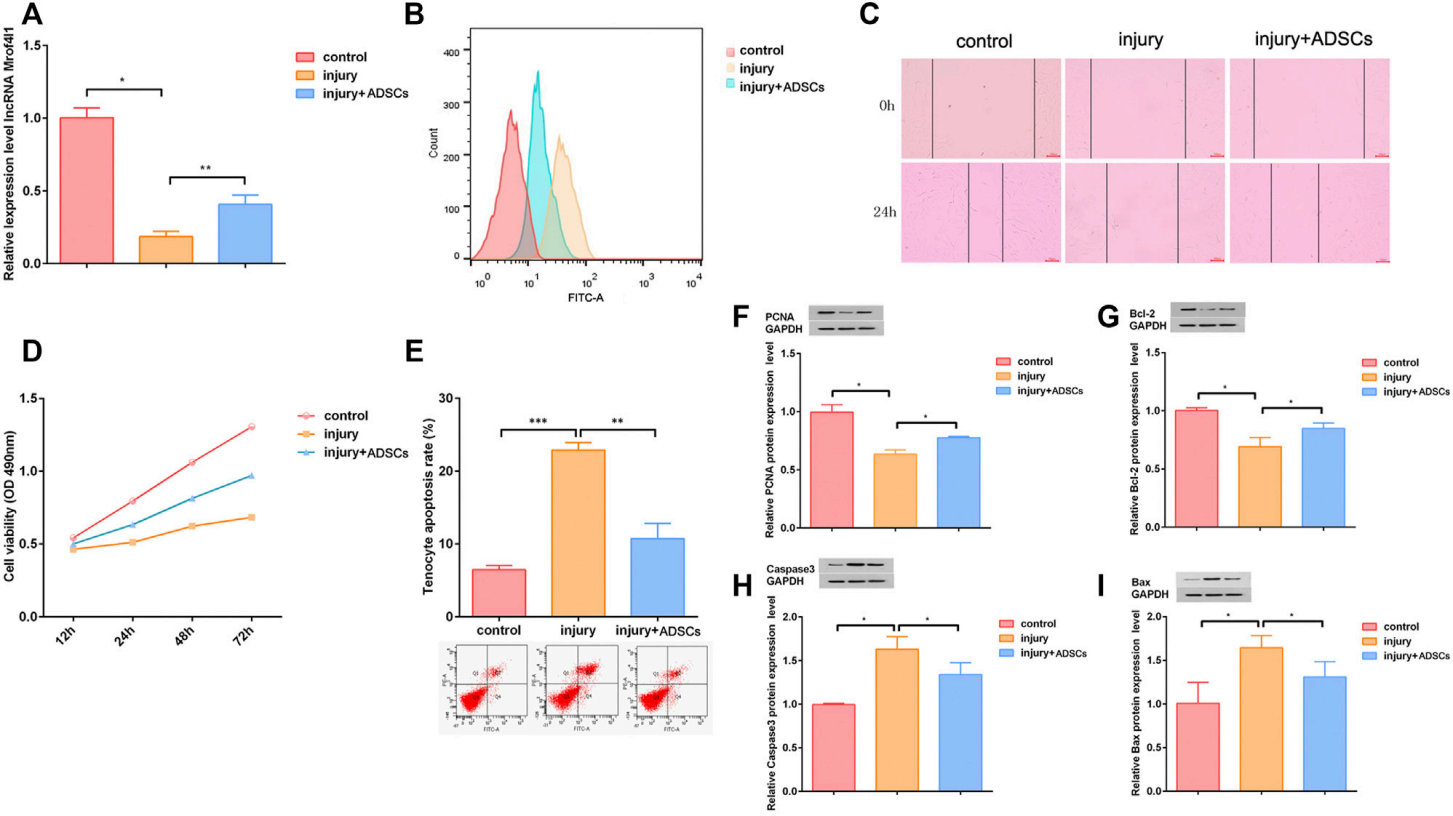
ADSCs rescued the tendon injury *in vitro*. **(A)** The expression of lncRNA Morf4l1 increased after co-culture of injured tenocytes and ADSCs **(B)** The oxidative stress level of injured tenocytes was reduced after co-culture **(C)** Wound-healing was used to detect the effect of co-culture on the migration ability of injured tenocytes; Scale:100 μm **(D)** The proliferation ability of injured tendinocytes increased after co-culture **(E)** The apoptosis level of injured tendinocytes decreased after co-culture **(F–I)** Western blot was used to detect the expression of PCNA, Bcl-2, Caspase3 and Bax of injured tenocytes after co-culture (*n* = 3; Mean ± SD, *p < 0.05, **p < 0.01, ***p < 0.001; Student’s t-tests).

Next, to verify the repair effect of ADSCs on injured tendon cells, ADSCs were co-cultured with injured tendon cells. The qRT-PCR assay results showed that lncRNA Morf4l1 expression was up-regulated after co-culture compared with injury group (Figure 4A). Flow cytometry analysis results showed that co-culture reduced oxidative stress in injured tenocytes (Figure 4B). Wound healing assay results illustrated that the co-culture significantly promoted the migration of injured tenocytes (Figure 4C). The CCK-8 results indicated that the proliferative ability of injured tenocytes was significantly promoted after co-culture (Figure 4D). The results of cell apoptosis assay by flow cytometry showed that the co-culture dramatically inhibited the apoptosis of injured tenocytes (Figure 4E). Western blot assay results displayed that the expression of Caspase3 and Bax was down-regulated were significantly up-regulated, whereas the expression of PCNA and Bcl-2 after co-culture (Figures 4F–I).

### ADSCs Promoted Tendon Wound Healing *in vivo*

**FIGURE 5:**
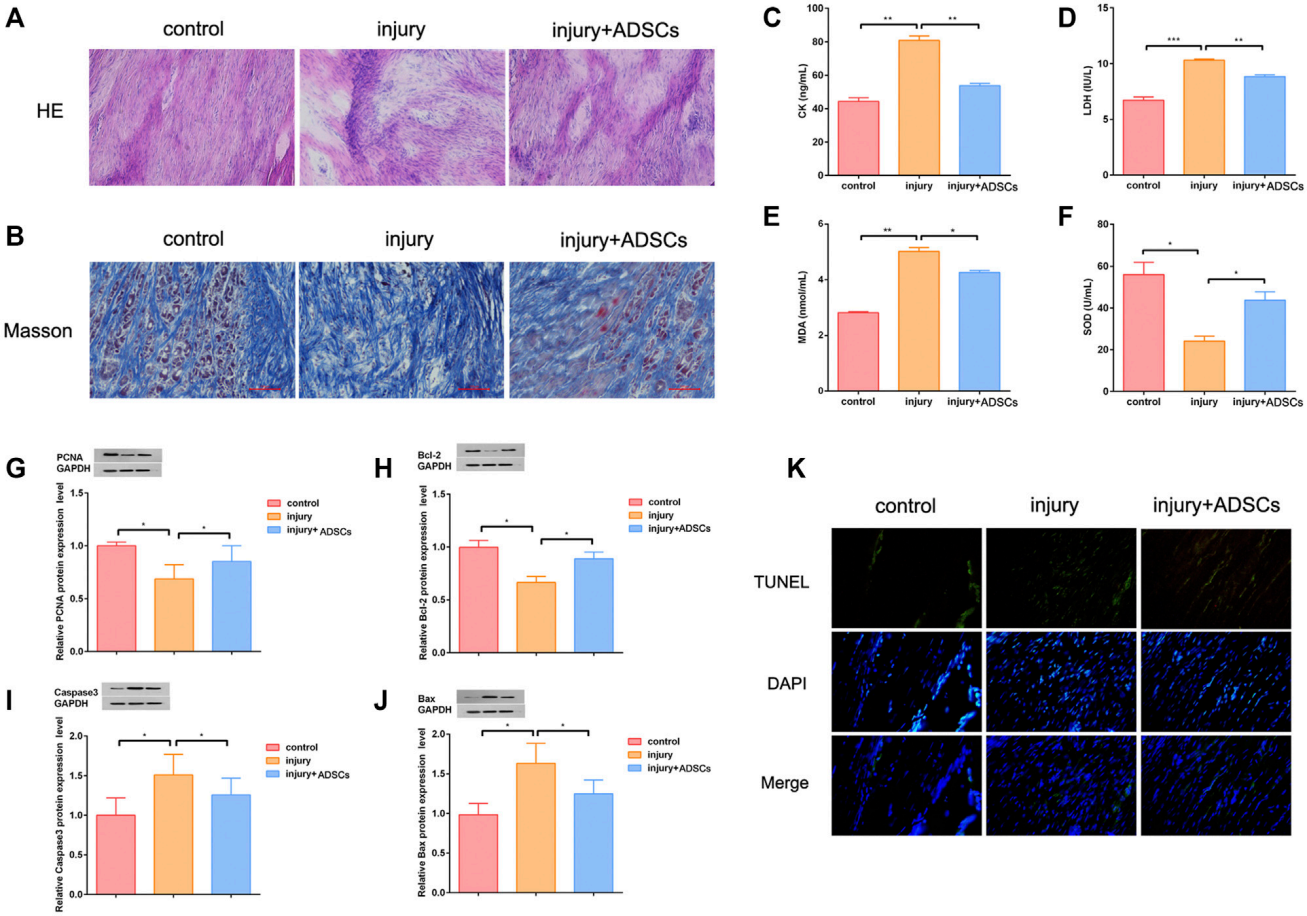
ADSCs promoted tendon wound healing *in vivo*. **(A)** HE staining was used to observe tendon tissue; Base magnification:×200 **(B)** Masson staining was used to observe tendon tissue; Scale:100 μm **(C–F)** ELISA assay was performed to detect the level of CK, LDH, MDA and SOD. The oxidative stress level of injured tendon was reduced after ADSCs treatment **(G–J)** Western blot was used to detect the expression of PCNA, Bcl-2, Caspase3 and Bax of injured tendon after ADSCs treatment **(K)** TUNEL assay showed that the level of apoptosis in tendon tissue was decreased after ADSCs treatment. The blue is the nucleus. the above green fluorescence intensity was positively correlated with the level of apoptosis; Under the merged image; Base magnification:×200 (*n* = 3; Mean ± SD, *p < 0.05, **p < 0.01, ***p < 0.001; Student’s t-tests).

Finally, ADSCs were injected into the injured tendon of rats to treat tendon injury. HE staining showed that after ADSCs treatment, the infiltrated inflammatory cells in the injured tendon tissue were reduced, tissue structure was improved, and tissue structural disorder had been significantly reduced (Figure 5A). Masson staining showed that after ADSCs treatment, collagen disorder had been significantly reduced (Figure 5B). ELISA results indicated that ADSCs treatment reduced oxidative stress in the injured tendon tissue (Figures 5C–F). Western blot assay results illustrated that the expression of PCNA and Bcl-2 were significantly up-regulated after ADSCs treatment, whereas the expression of Caspase3 and Bax were down-regulated (Figures 5G–J). The results of TUNEL apoptosis assay showed that the apoptosis level of injured tendon tissues was decreased after ADSCs treatment (Figure 5K).

## Discussion

Acute tendon injuries often occur during sports and other strenuous activities. The naturally healed tendon often has inferior mechanical properties and is prone to re-injury (Boyer et al., 2005; Sharma and Maffulli, 2006). To date, the repair of tendon injuries has been a huge challenge. LncRNAs have been reported to be associated with a variety of diseases. For example, LncRNAuc.134 inhibits the progression of hepatocellular carcinoma by inhibiting CUL4A-mediated LATS1 ubiquitination (Ni et al., 2017). Knockdown of lncRNA KCNQ1OT1 inhibited the adipogenic and osteogenic differentiation of TSCs (Yu et al., 2018a). In this study, we identified a number of lncRNAs that were aberrantly expressed in damaged tendon tissue. Among them, lncRNA Morf4l1 was significantly down-regulated in injured tendon tissue. Here we speculated that lncRNA Morf4l1 might be a key target in tendon repair. In addition, lncRNAs is the research hotspot in the field of epigenetics. LncRNAs can be divided into antisense, antisense, intergene, overlap, intron and full overlap according to their relative gene locations (Maruyama and Suzuki, 2012). All types of lncRNA can regulate their target molecules at the pre-transcriptional, transcriptional or post-transcriptional level by binding to DNA, RNA or protein. Previously, there have been a few reports of differential promoter methylation of Mmp25, Foxf1, Leprel2, Igfbp6 and Peg12 in tendinopathy through genome-wide analysis (Trella et al., 2017). And there are few reports available on the mechanism of lncRNA promoter methylation and tendon repair. LncRNA in different subcellular localization, corresponding modification methods are different. Figures 1C,D showed that lncRNA Morf4l1 was mainly located in the nucleus. In the nucleus, lncRNA often undergoes epigenetic modification and affects the expression level (Huarte et al., 2010; Simon et al., 2013; Ma et al., 2020). Pyrosequencing and qRT-PCR also confirmed promoter hypermethylation of lncRNA Morf4l1 in injured tendon cells, thus leading to down-regulation of expression.

We then used demethylation drugs to evaluate the effect of lncRNA promoter methylation on injured tendon cells, and found that after lncRNA demethylation, the oxidative stress level of tenocytes was decreased and the cell proliferation ability was increased. This suggested that lncRNA demethylation saved the tendon injury. Subsequently, we predicted and verified the targeted genes of lncRNA Morf4l1, which was found to promote the expression of TGF-β2 through targeting 3′U of miR-145-5p. Although miR-145-5p is rarely reported in tendinopathy, it still shows its inhibitory ability. LncRNA-PCAT1 sponges miR-145-5p and promote TLRA-associated hADSCs osteogenic differentiation by activating toll-like receptor signaling pathways (Yu et al., 2018b). There were also reports that circPTN sponged miR-145-5p to promote proliferation and stemness in glioma (Chen et al., 2019). The TGF-β pathway is one of the most identified signaling pathways for tendon development (Liu et al., 2017), which could promote proliferation, migration and fibrotic activity in rotator cuff tenocytes (Li et al., 2020). Overall, our results foreshadowed that lncRNA Morf4l1 promoted the expression of TGF-β2 through targeting 3′U of miR-145-5p, thereby promoting tendon repair.

MSCs are an ideal cell source for tissue regeneration that are present in almost all tissues, including bone marrow, adipose, and synovium, and are easily extracted (Ding et al., 2006; Ding et al., 2007; Ding et al., 2011). In particular, the frequency and yield of ADSCs were about 2500 times higher than that of BM-MSCs (Fraser et al., 2008; Baer and Geiger, 2012). There was evidence that ADSCs play a key role in maintaining skin tissue structure, even as a physiological response to local damage, or as a mechanism for rejuvenation by seeding younger cells onto the outside of the *epidermis* (Vishnubalaji et al., 2012; Marfia et al., 2015; Zhang et al., 2017). Studies have shown that MSCs play an important role in methylation involved in cell survival (Marofi et al., 2022). Qiu et al. reported that exosomes secreted by curcumin-treated MSCs reduced DNA methylation of miR-143 and miR-124 promoters and alleviated osteoarthritis (Qiu et al., 2020). In this study, we found that the expression of lncRNA Morf4l1 was up-regulated after co-culture of ADSCs and injured tenocytes. ADSCs treatment inhibit oxidative stress and promote the proliferation of tendon cells *in vivo*. *In vitro*, ADSCs treatment also inhibit oxidative stress and reduce the disorder of collagen repair damage. This result is consistent with the use of demethylation drugs. Therefore, we believe that ADSCs promote tendon repair by rescuing the methylation-induced decreased expression of lncRNA Morf4l1. However, this study lacked direct evidence that ADSC reduced the methylation level of lncRNA Morf4l1, which was the limitation of this study. In future experiments, we will focus on how to solve this problem better.

In conclusion, we confirmed that lncRNA Morf4l1 methylation inhibited tenocyte proliferation *in vitro*. ADSCs alleviated methylation to promote the proliferation of tenocytes and repair injured tendons, while providing a new target for tendon therapy.

## Data Availability

The sequencing data mentioned in this paper have been uploaded to GEO database. Its serial number is GSE206380.
