# Supplementary figures and images for: ADSCs Promote Tenocyte Proliferation by Reducing the Methylation Level of lncRNA Morf4l1 in Tendon Injury
Source: Front Chem. 2022 Jul 4;10:908312. doi: 10.3389/fchem.2022.908312 (PMC9290323; doi:10.3389/fchem.2022.908312)

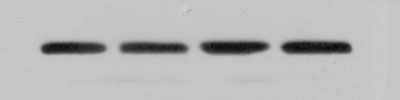

Supplement: Supplementary file 1 [file DataSheet3.ZIP › Fig. 3 LncRNA Morf4l1 promoted the expression of TGF-a┬2 through targeting 3'U of miR-145-5p/Fig.3(I) WB/GAPDH(1).jpg]

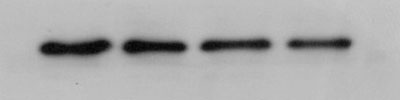

Supplement: Supplementary file 1 [file DataSheet3.ZIP › Fig. 3 LncRNA Morf4l1 promoted the expression of TGF-a┬2 through targeting 3'U of miR-145-5p/Fig.3(I) WB/GAPDH(2).jpg]

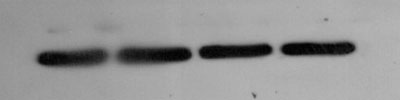

Supplement: Supplementary file 1 [file DataSheet3.ZIP › Fig. 3 LncRNA Morf4l1 promoted the expression of TGF-a┬2 through targeting 3'U of miR-145-5p/Fig.3(I) WB/GAPDH(3).jpg]

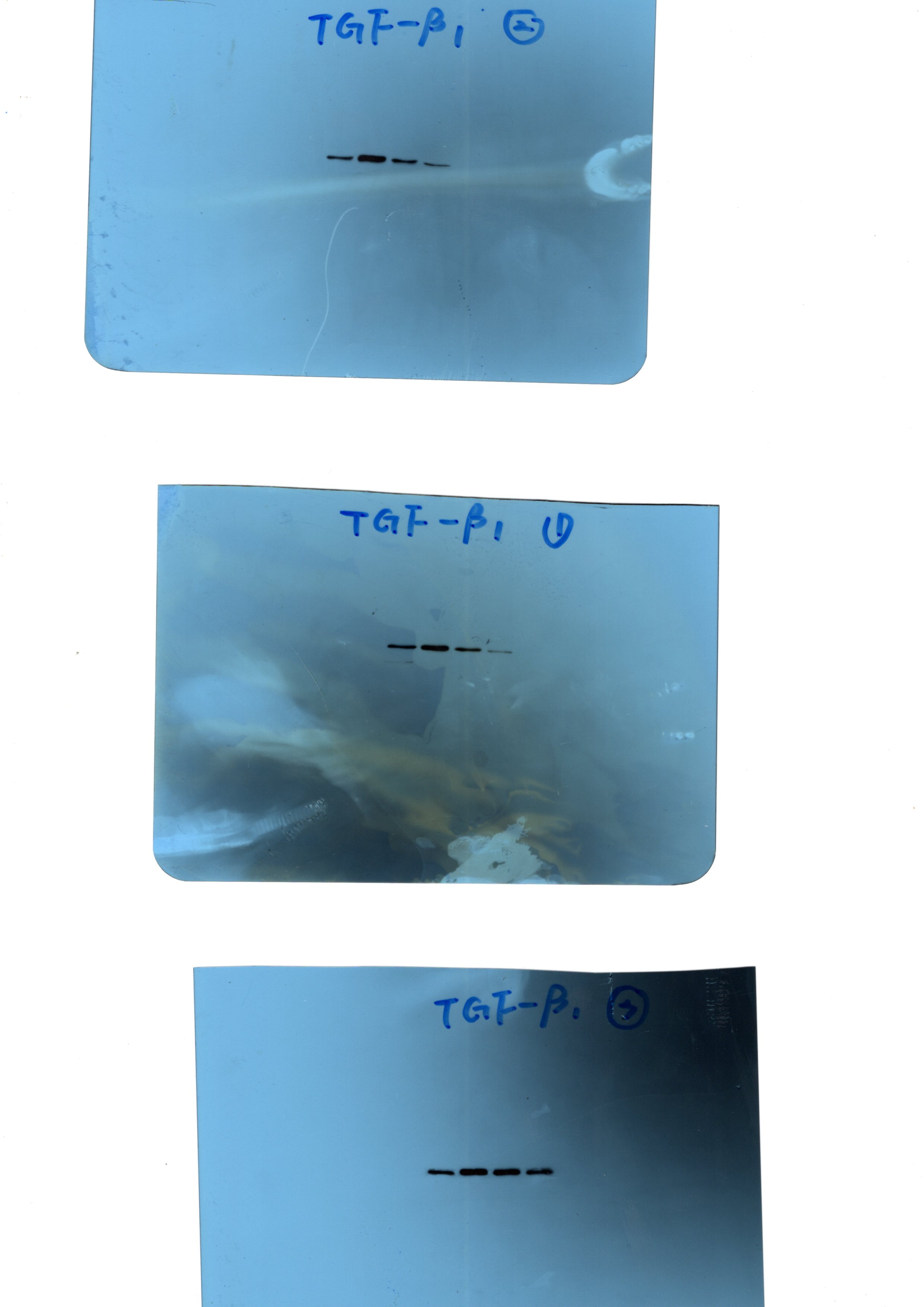

Supplement: Supplementary file 1 [file DataSheet3.ZIP › Fig. 3 LncRNA Morf4l1 promoted the expression of TGF-a┬2 through targeting 3'U of miR-145-5p/Fig.3(I) WB/img872.jpg]

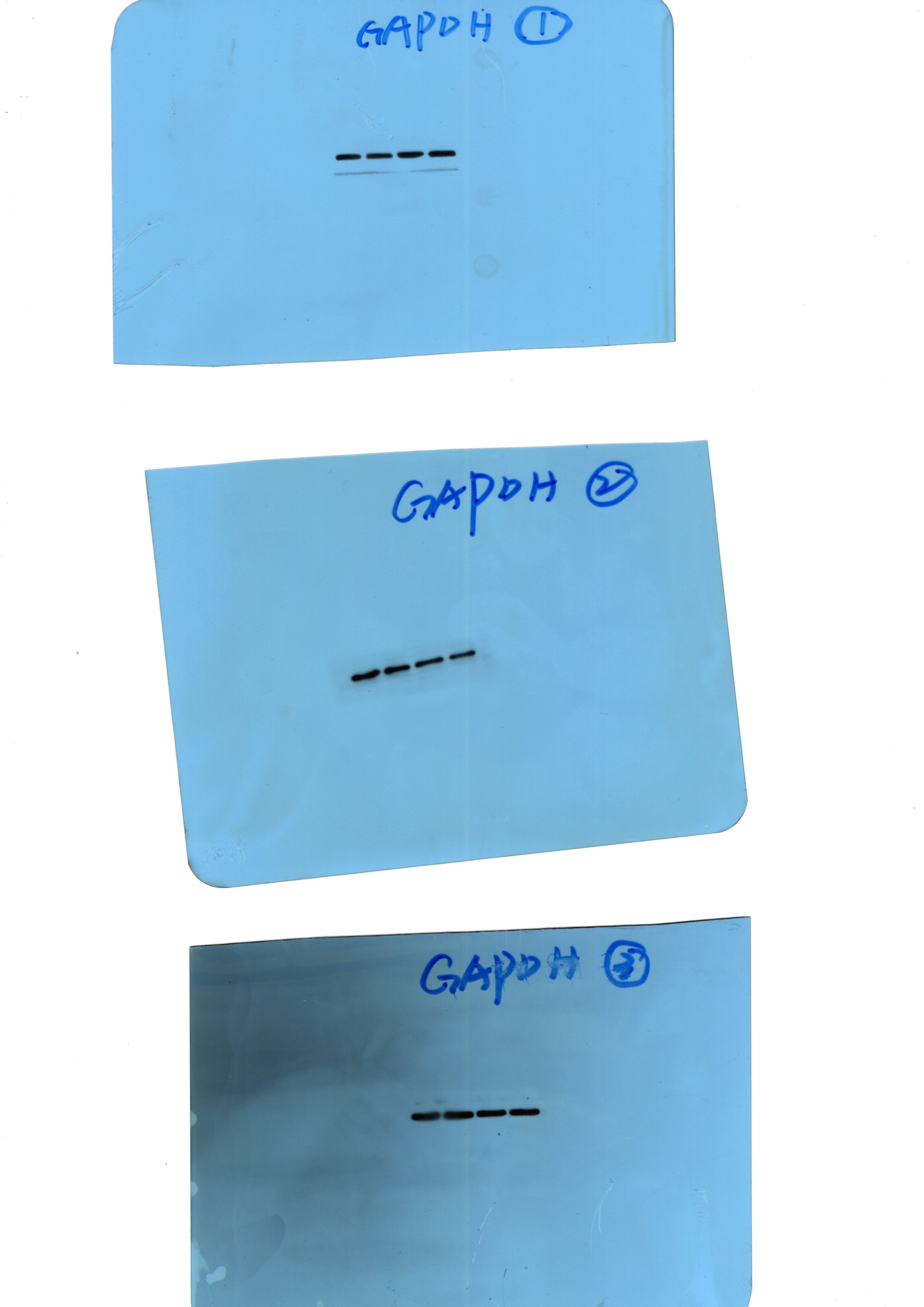

Supplement: Supplementary file 1 [file DataSheet3.ZIP › Fig. 3 LncRNA Morf4l1 promoted the expression of TGF-a┬2 through targeting 3'U of miR-145-5p/Fig.3(I) WB/img873.jpg]

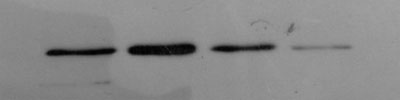

Supplement: Supplementary file 1 [file DataSheet3.ZIP › Fig. 3 LncRNA Morf4l1 promoted the expression of TGF-a┬2 through targeting 3'U of miR-145-5p/Fig.3(I) WB/TGF-aó1(2).jpg]

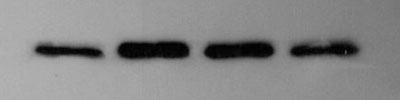

Supplement: Supplementary file 1 [file DataSheet3.ZIP › Fig. 3 LncRNA Morf4l1 promoted the expression of TGF-a┬2 through targeting 3'U of miR-145-5p/Fig.3(I) WB/TGF-aó1(3).jpg]

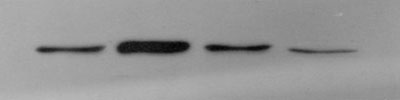

Supplement: Supplementary file 1 [file DataSheet3.ZIP › Fig. 3 LncRNA Morf4l1 promoted the expression of TGF-a┬2 through targeting 3'U of miR-145-5p/Fig.3(I) WB/TGF-aó1.jpg]

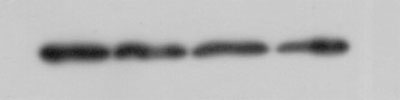

Supplement: Supplementary file 1 [file DataSheet3.ZIP › Fig. 3 LncRNA Morf4l1 promoted the expression of TGF-a┬2 through targeting 3'U of miR-145-5p/Fig3.(F) WB/GAPDH(1).jpg]

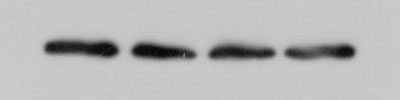

Supplement: Supplementary file 1 [file DataSheet3.ZIP › Fig. 3 LncRNA Morf4l1 promoted the expression of TGF-a┬2 through targeting 3'U of miR-145-5p/Fig3.(F) WB/GAPDH(2).jpg]

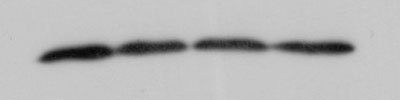

Supplement: Supplementary file 1 [file DataSheet3.ZIP › Fig. 3 LncRNA Morf4l1 promoted the expression of TGF-a┬2 through targeting 3'U of miR-145-5p/Fig3.(F) WB/GAPDH(3).jpg]

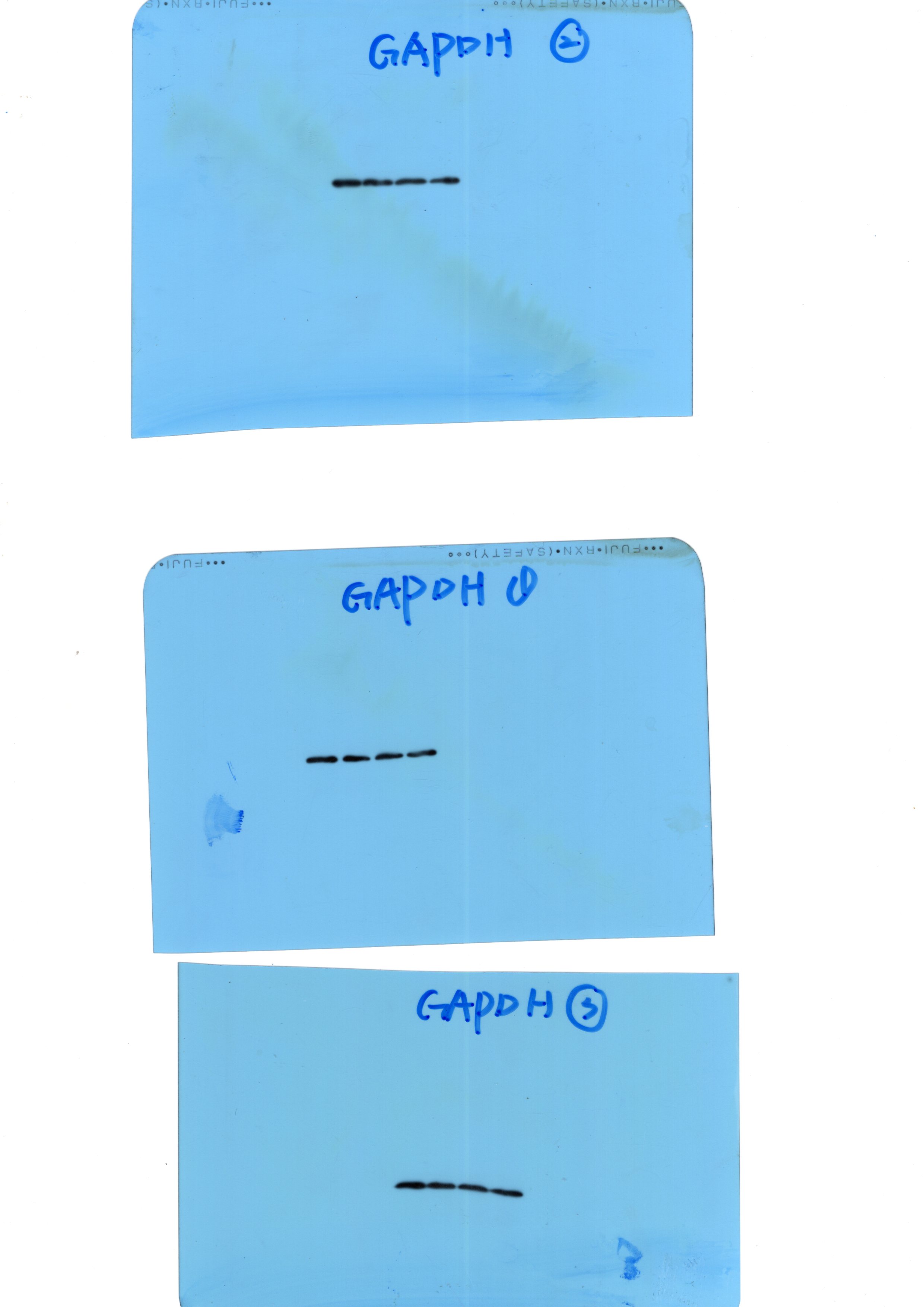

Supplement: Supplementary file 1 [file DataSheet3.ZIP › Fig. 3 LncRNA Morf4l1 promoted the expression of TGF-a┬2 through targeting 3'U of miR-145-5p/Fig3.(F) WB/img870.jpg]

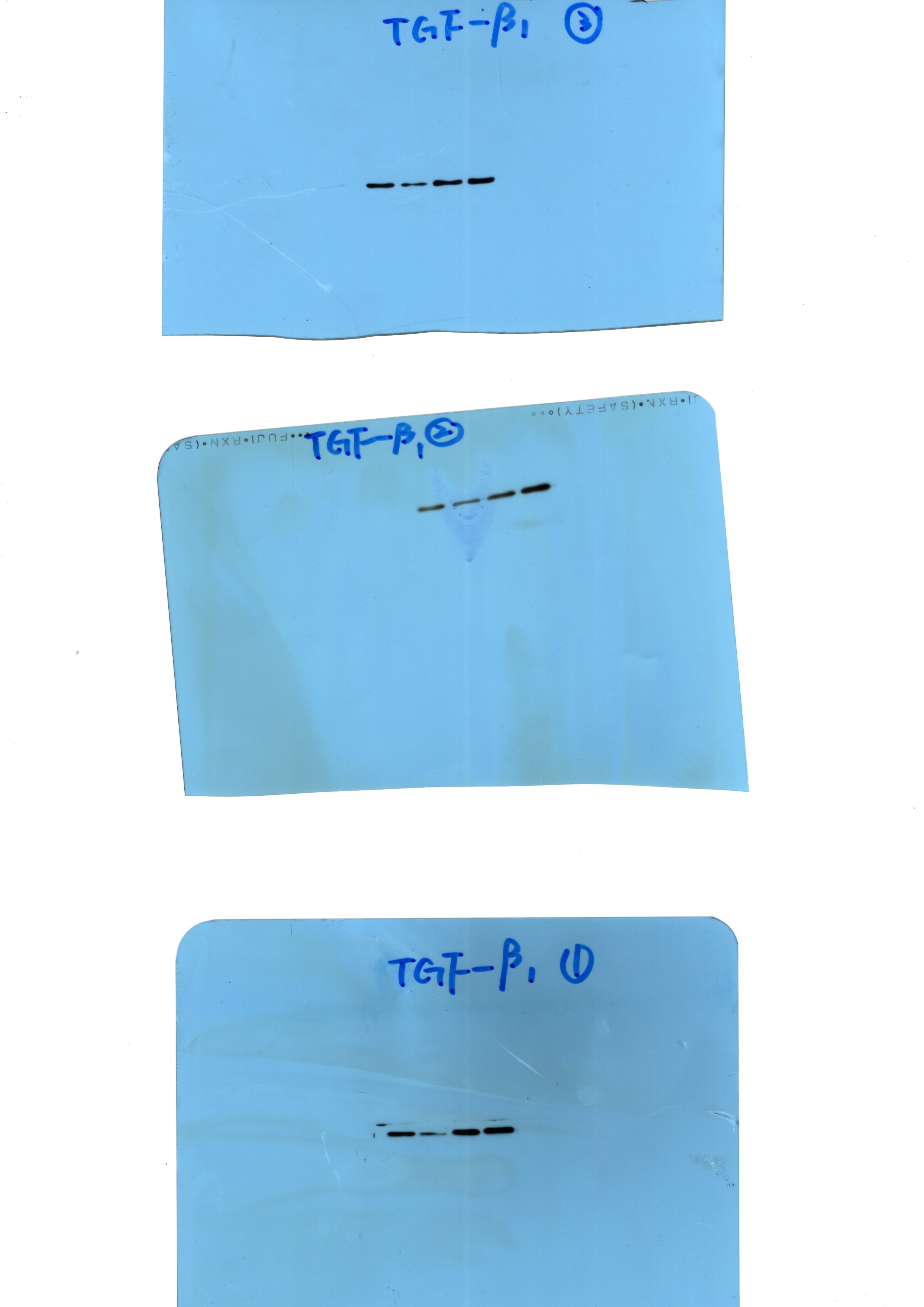

Supplement: Supplementary file 1 [file DataSheet3.ZIP › Fig. 3 LncRNA Morf4l1 promoted the expression of TGF-a┬2 through targeting 3'U of miR-145-5p/Fig3.(F) WB/img871.jpg]

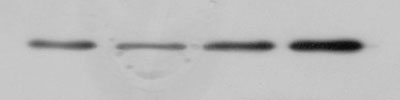

Supplement: Supplementary file 1 [file DataSheet3.ZIP › Fig. 3 LncRNA Morf4l1 promoted the expression of TGF-a┬2 through targeting 3'U of miR-145-5p/Fig3.(F) WB/TGF-a┬1ú¿2ú⌐.jpg]

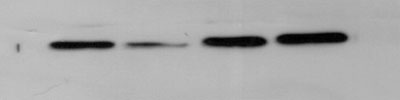

Supplement: Supplementary file 1 [file DataSheet3.ZIP › Fig. 3 LncRNA Morf4l1 promoted the expression of TGF-a┬2 through targeting 3'U of miR-145-5p/Fig3.(F) WB/TGF-a┬1ú¿3ú⌐.jpg]

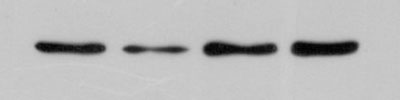

Supplement: Supplementary file 1 [file DataSheet3.ZIP › Fig. 3 LncRNA Morf4l1 promoted the expression of TGF-a┬2 through targeting 3'U of miR-145-5p/Fig3.(F) WB/TGF-a┬1.jpg]

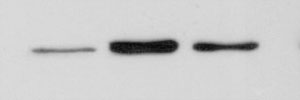

Supplement: Supplementary file 2 [file DataSheet4.ZIP › Fig. 4 ADSCs rescued the tendon injury in vitro/Fig.4(F)WB/Bax(1).jpg]

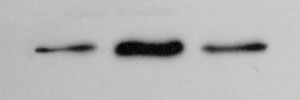

Supplement: Supplementary file 2 [file DataSheet4.ZIP › Fig. 4 ADSCs rescued the tendon injury in vitro/Fig.4(F)WB/Bax(2).jpg]

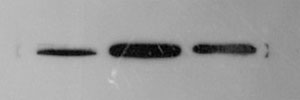

Supplement: Supplementary file 2 [file DataSheet4.ZIP › Fig. 4 ADSCs rescued the tendon injury in vitro/Fig.4(F)WB/Bax(3).jpg]

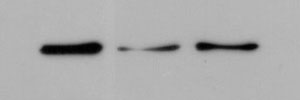

Supplement: Supplementary file 2 [file DataSheet4.ZIP › Fig. 4 ADSCs rescued the tendon injury in vitro/Fig.4(F)WB/BCL-2(1).jpg]

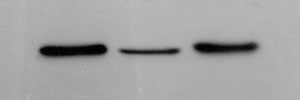

Supplement: Supplementary file 2 [file DataSheet4.ZIP › Fig. 4 ADSCs rescued the tendon injury in vitro/Fig.4(F)WB/BCL-2(2).jpg]

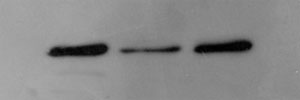

Supplement: Supplementary file 2 [file DataSheet4.ZIP › Fig. 4 ADSCs rescued the tendon injury in vitro/Fig.4(F)WB/BCL-2(3).jpg]

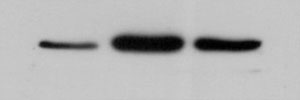

Supplement: Supplementary file 2 [file DataSheet4.ZIP › Fig. 4 ADSCs rescued the tendon injury in vitro/Fig.4(F)WB/Caspase3(2).jpg]

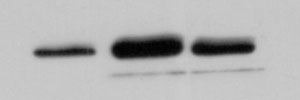

Supplement: Supplementary file 2 [file DataSheet4.ZIP › Fig. 4 ADSCs rescued the tendon injury in vitro/Fig.4(F)WB/Caspase3(3).jpg]

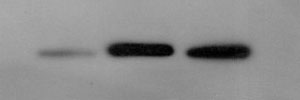

Supplement: Supplementary file 2 [file DataSheet4.ZIP › Fig. 4 ADSCs rescued the tendon injury in vitro/Fig.4(F)WB/Caspase3.jpg]

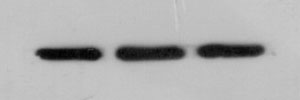

Supplement: Supplementary file 2 [file DataSheet4.ZIP › Fig. 4 ADSCs rescued the tendon injury in vitro/Fig.4(F)WB/GAPDH(1).jpg]

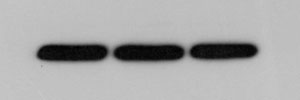

Supplement: Supplementary file 2 [file DataSheet4.ZIP › Fig. 4 ADSCs rescued the tendon injury in vitro/Fig.4(F)WB/GAPDH(2).jpg]

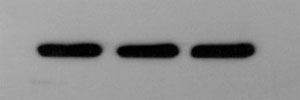

Supplement: Supplementary file 2 [file DataSheet4.ZIP › Fig. 4 ADSCs rescued the tendon injury in vitro/Fig.4(F)WB/GAPDH(3).jpg]

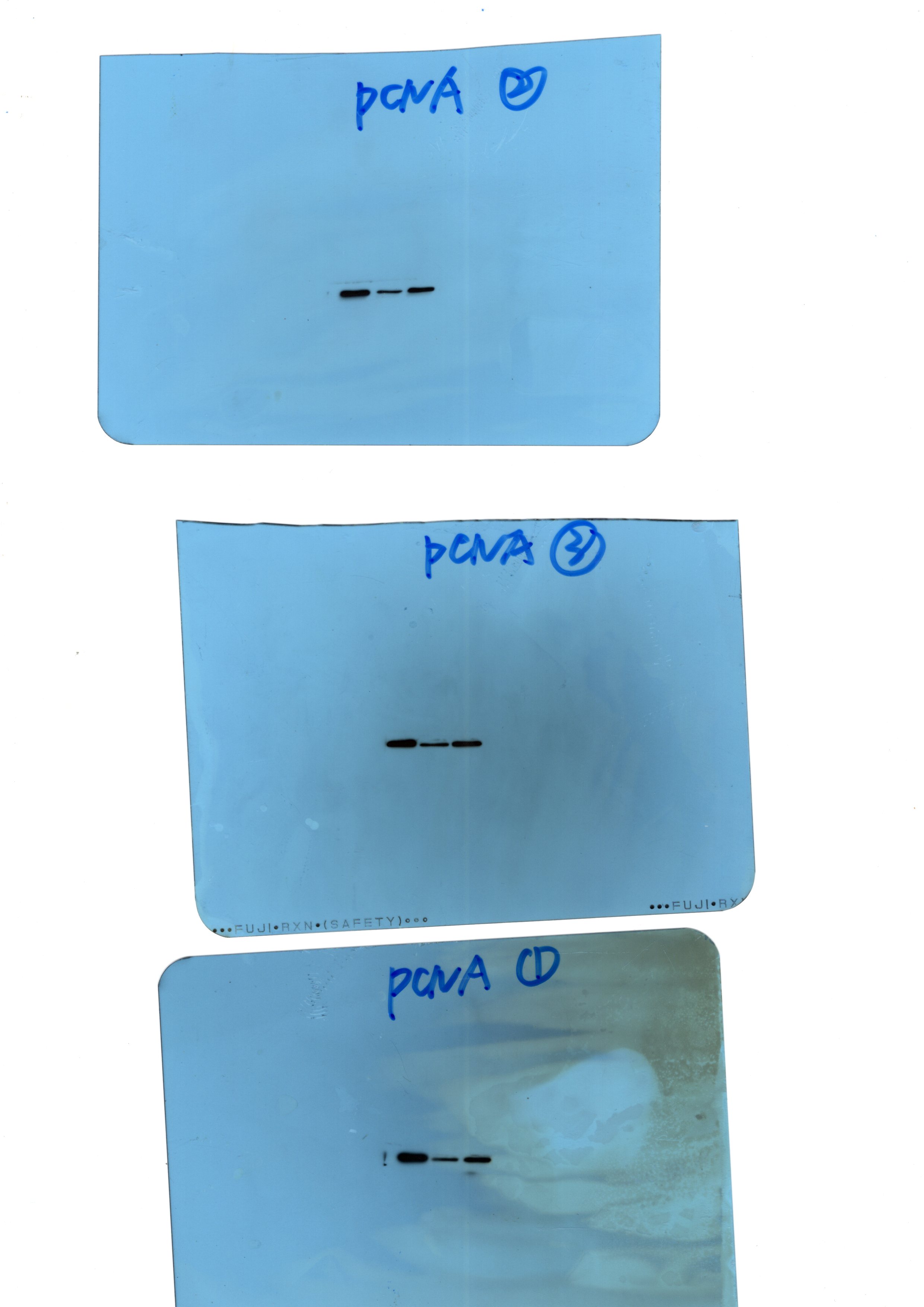

Supplement: Supplementary file 2 [file DataSheet4.ZIP › Fig. 4 ADSCs rescued the tendon injury in vitro/Fig.4(F)WB/img874.jpg]

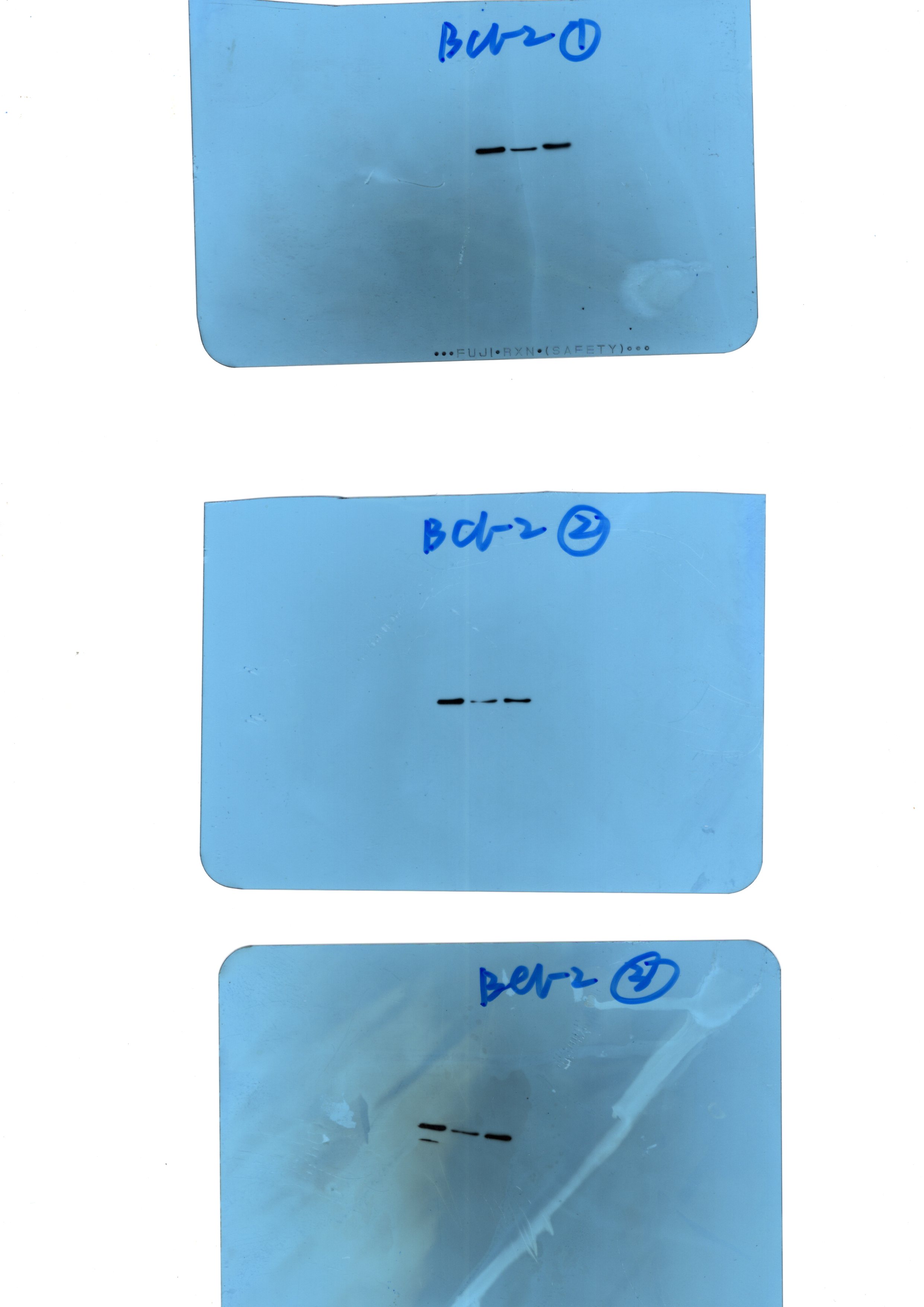

Supplement: Supplementary file 2 [file DataSheet4.ZIP › Fig. 4 ADSCs rescued the tendon injury in vitro/Fig.4(F)WB/img875.jpg]

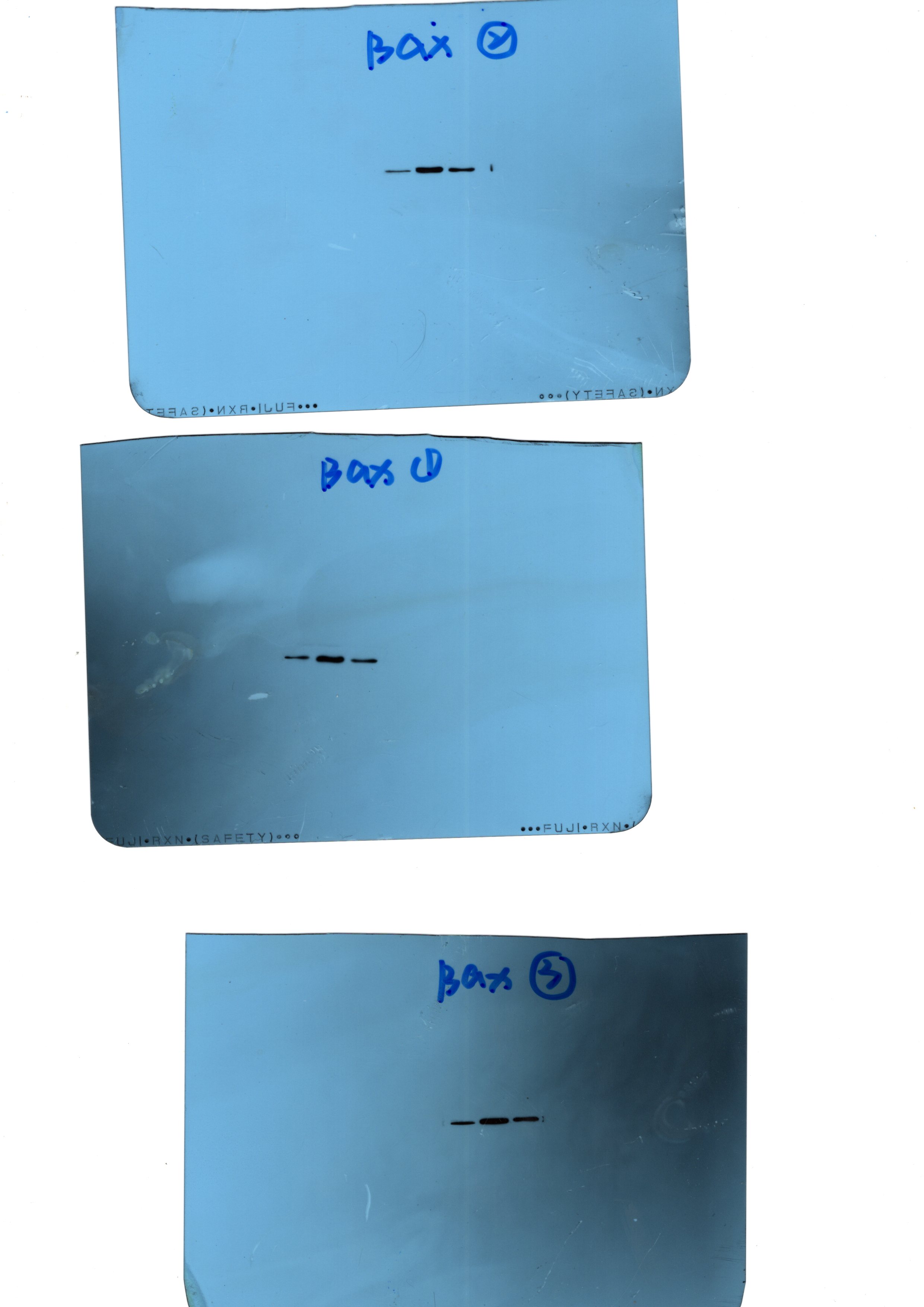

Supplement: Supplementary file 2 [file DataSheet4.ZIP › Fig. 4 ADSCs rescued the tendon injury in vitro/Fig.4(F)WB/img876.jpg]

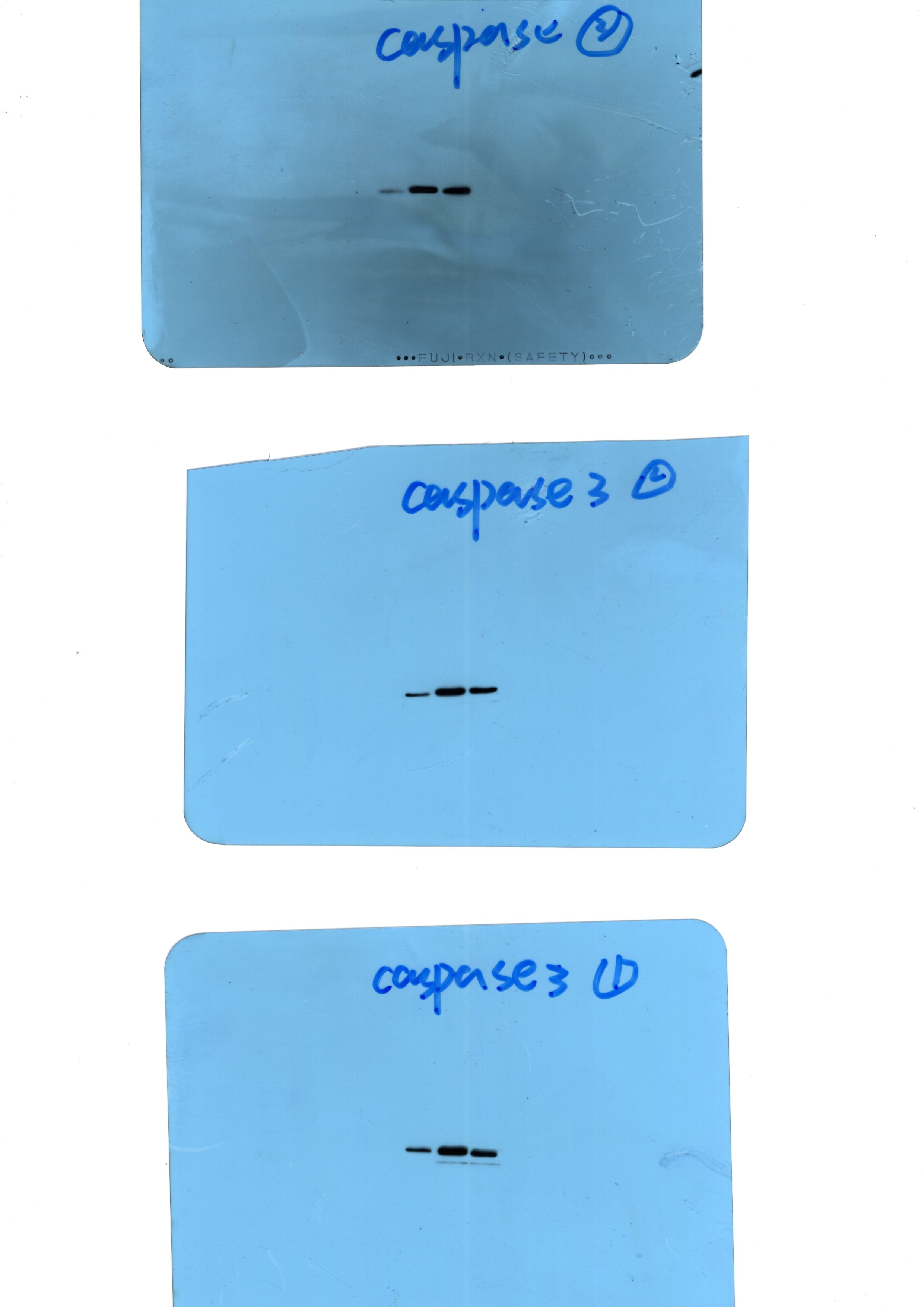

Supplement: Supplementary file 2 [file DataSheet4.ZIP › Fig. 4 ADSCs rescued the tendon injury in vitro/Fig.4(F)WB/img877.jpg]

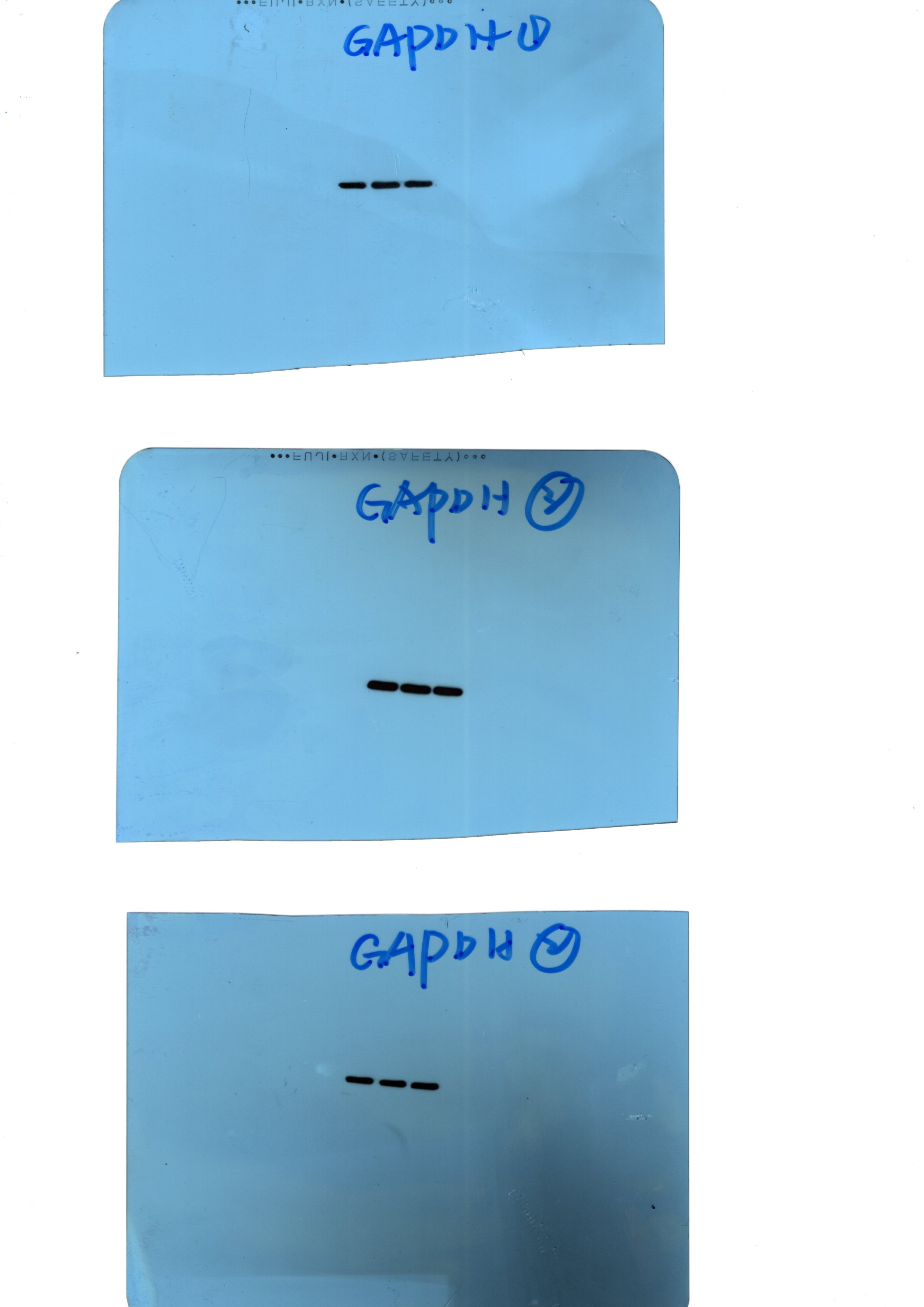

Supplement: Supplementary file 2 [file DataSheet4.ZIP › Fig. 4 ADSCs rescued the tendon injury in vitro/Fig.4(F)WB/img878.jpg]

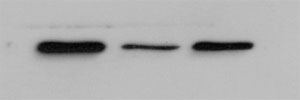

Supplement: Supplementary file 2 [file DataSheet4.ZIP › Fig. 4 ADSCs rescued the tendon injury in vitro/Fig.4(F)WB/PCNA(1).jpg]

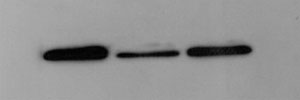

Supplement: Supplementary file 2 [file DataSheet4.ZIP › Fig. 4 ADSCs rescued the tendon injury in vitro/Fig.4(F)WB/PCNA(2).jpg]

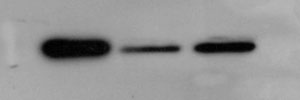

Supplement: Supplementary file 2 [file DataSheet4.ZIP › Fig. 4 ADSCs rescued the tendon injury in vitro/Fig.4(F)WB/PCNA(3).jpg]

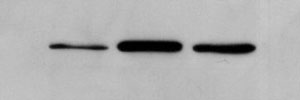

Supplement: Supplementary file 4 [file DataSheet2.ZIP › Fig. 2 LncRNA Morf4l1 inhibited oxidative stress and promoted proliferation of tenocytes/Fig.2(E)WB/BAX(1).jpg]

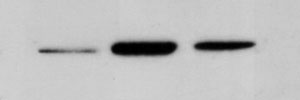

Supplement: Supplementary file 4 [file DataSheet2.ZIP › Fig. 2 LncRNA Morf4l1 inhibited oxidative stress and promoted proliferation of tenocytes/Fig.2(E)WB/BAX(2).jpg]

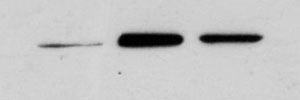

Supplement: Supplementary file 4 [file DataSheet2.ZIP › Fig. 2 LncRNA Morf4l1 inhibited oxidative stress and promoted proliferation of tenocytes/Fig.2(E)WB/BAX(3).jpg]

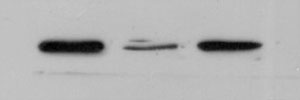

Supplement: Supplementary file 4 [file DataSheet2.ZIP › Fig. 2 LncRNA Morf4l1 inhibited oxidative stress and promoted proliferation of tenocytes/Fig.2(E)WB/BCL-2(1).jpg]

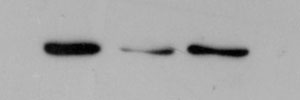

Supplement: Supplementary file 4 [file DataSheet2.ZIP › Fig. 2 LncRNA Morf4l1 inhibited oxidative stress and promoted proliferation of tenocytes/Fig.2(E)WB/BCL-2(2).jpg]

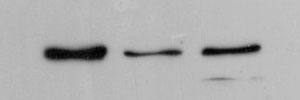

Supplement: Supplementary file 4 [file DataSheet2.ZIP › Fig. 2 LncRNA Morf4l1 inhibited oxidative stress and promoted proliferation of tenocytes/Fig.2(E)WB/BCL-2(3).jpg]

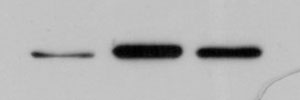

Supplement: Supplementary file 4 [file DataSheet2.ZIP › Fig. 2 LncRNA Morf4l1 inhibited oxidative stress and promoted proliferation of tenocytes/Fig.2(E)WB/Caspase3(2).jpg]

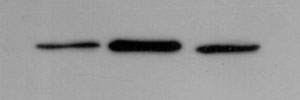

Supplement: Supplementary file 4 [file DataSheet2.ZIP › Fig. 2 LncRNA Morf4l1 inhibited oxidative stress and promoted proliferation of tenocytes/Fig.2(E)WB/Caspase3(3).jpg]

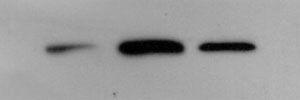

Supplement: Supplementary file 4 [file DataSheet2.ZIP › Fig. 2 LncRNA Morf4l1 inhibited oxidative stress and promoted proliferation of tenocytes/Fig.2(E)WB/Caspase3.jpg]

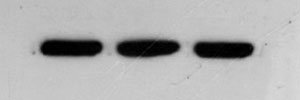

Supplement: Supplementary file 4 [file DataSheet2.ZIP › Fig. 2 LncRNA Morf4l1 inhibited oxidative stress and promoted proliferation of tenocytes/Fig.2(E)WB/GAPDH(1).jpg]

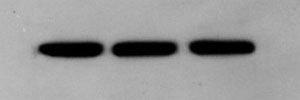

Supplement: Supplementary file 4 [file DataSheet2.ZIP › Fig. 2 LncRNA Morf4l1 inhibited oxidative stress and promoted proliferation of tenocytes/Fig.2(E)WB/GAPDH(2).jpg]

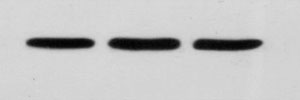

Supplement: Supplementary file 4 [file DataSheet2.ZIP › Fig. 2 LncRNA Morf4l1 inhibited oxidative stress and promoted proliferation of tenocytes/Fig.2(E)WB/GAPDH(3).jpg]

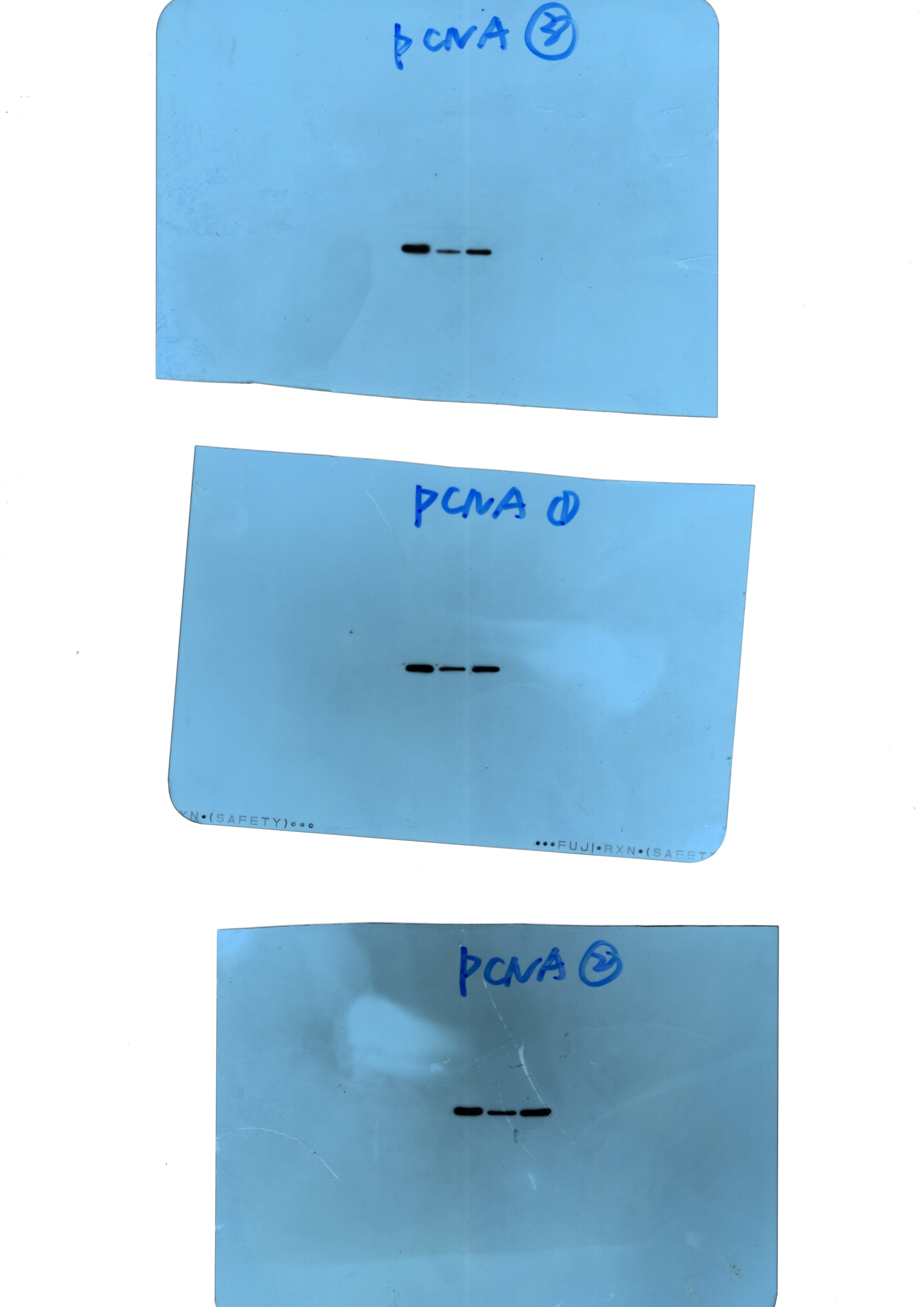

Supplement: Supplementary file 4 [file DataSheet2.ZIP › Fig. 2 LncRNA Morf4l1 inhibited oxidative stress and promoted proliferation of tenocytes/Fig.2(E)WB/img865.jpg]

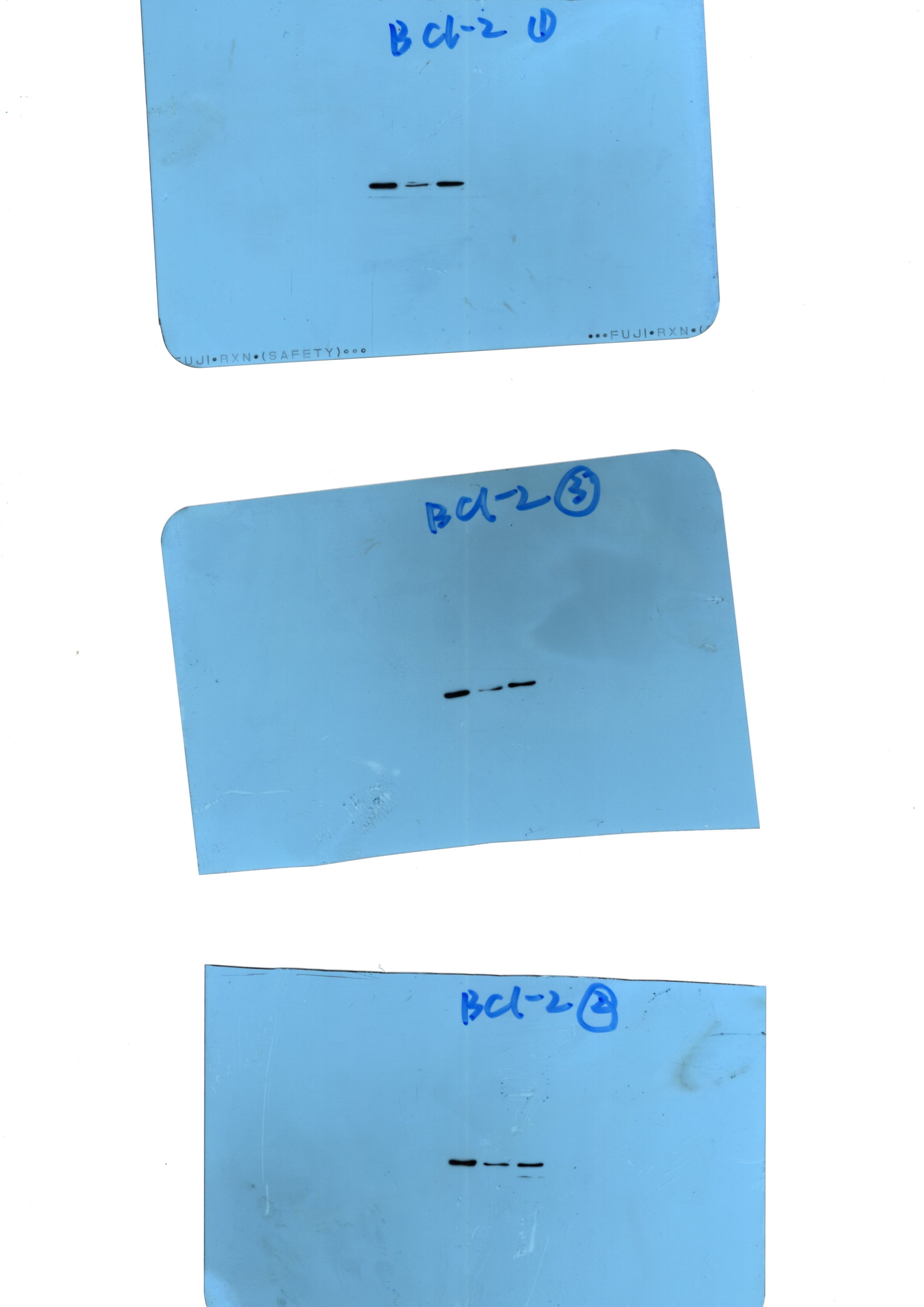

Supplement: Supplementary file 4 [file DataSheet2.ZIP › Fig. 2 LncRNA Morf4l1 inhibited oxidative stress and promoted proliferation of tenocytes/Fig.2(E)WB/img866.jpg]

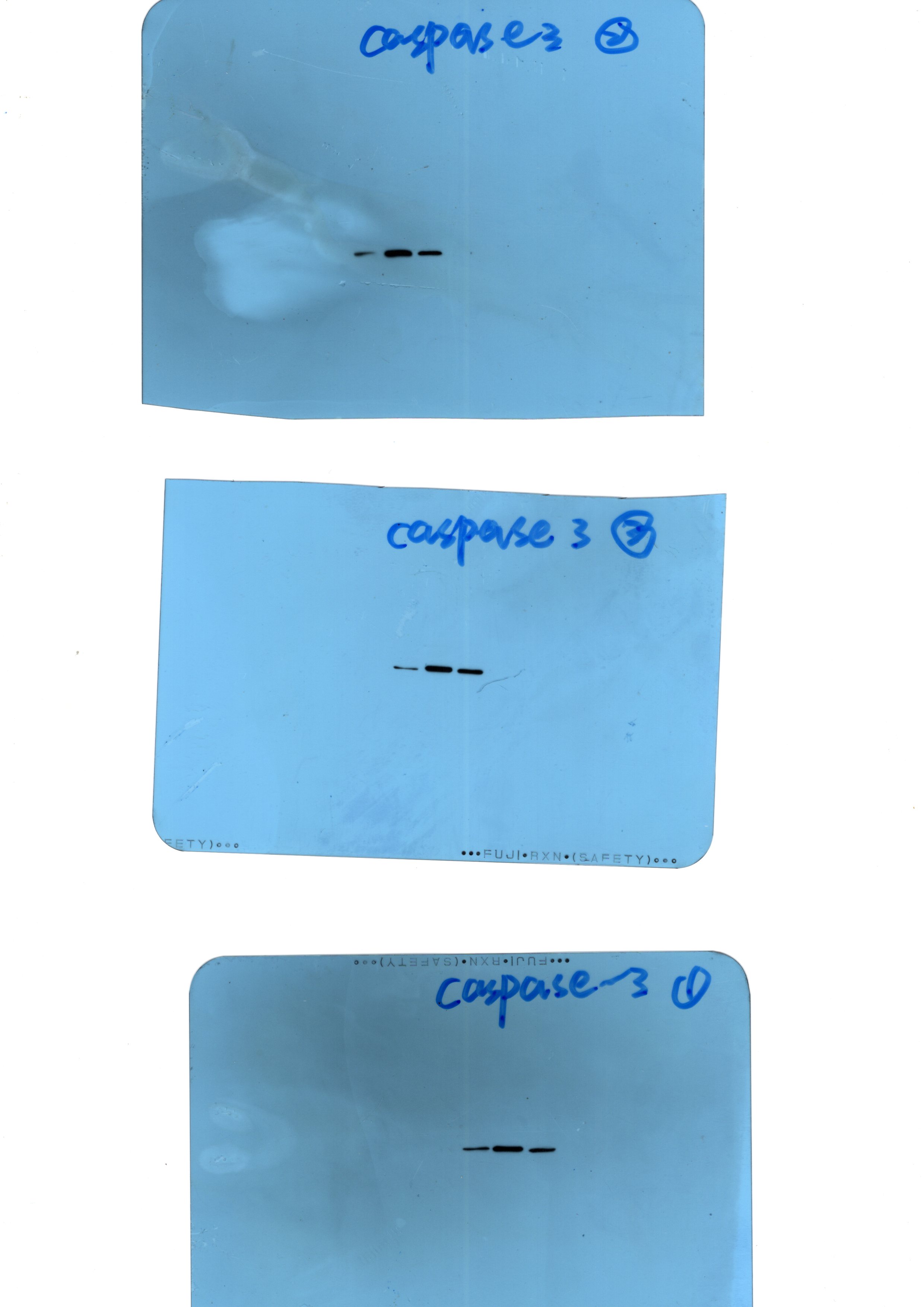

Supplement: Supplementary file 4 [file DataSheet2.ZIP › Fig. 2 LncRNA Morf4l1 inhibited oxidative stress and promoted proliferation of tenocytes/Fig.2(E)WB/img867.jpg]

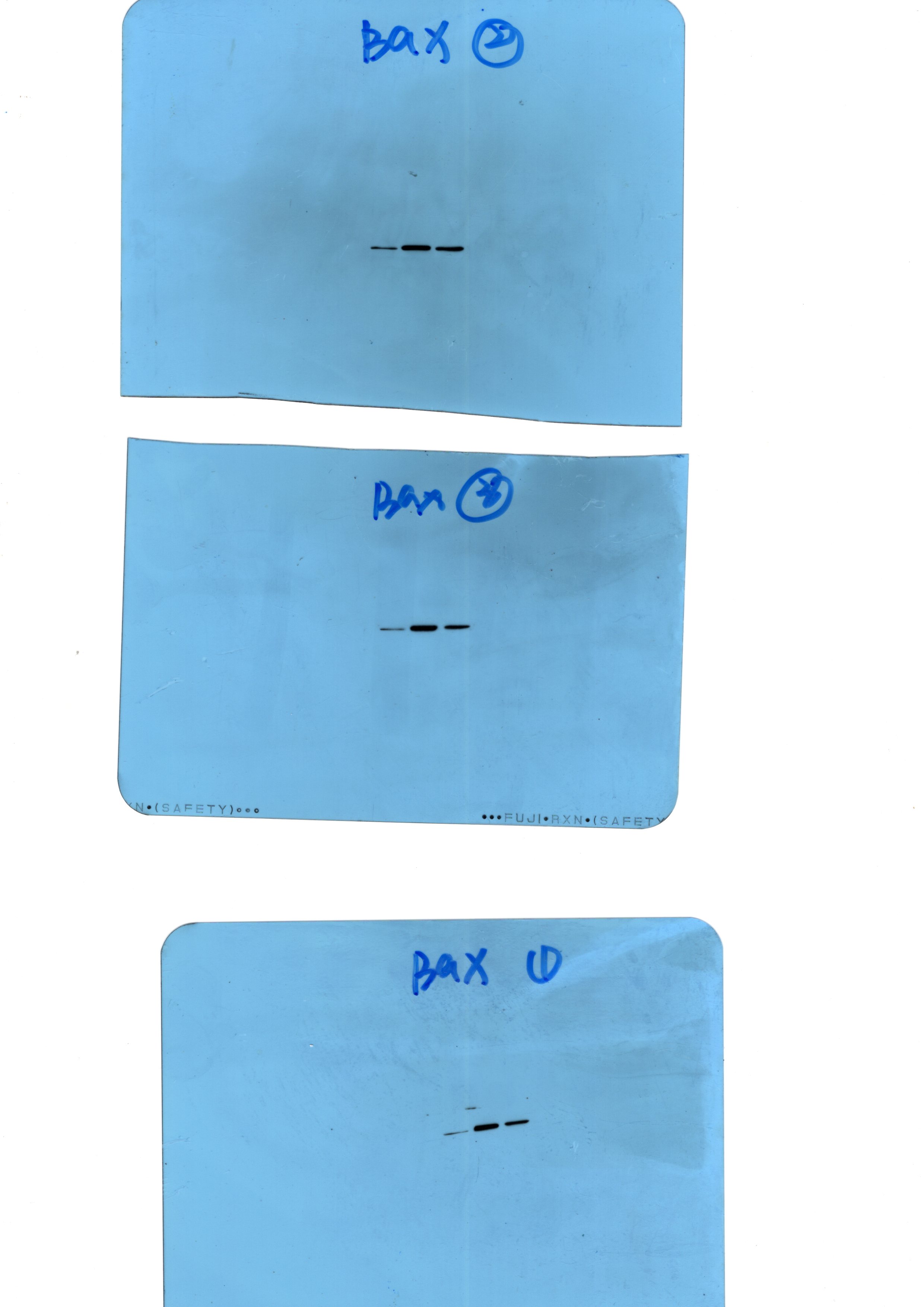

Supplement: Supplementary file 4 [file DataSheet2.ZIP › Fig. 2 LncRNA Morf4l1 inhibited oxidative stress and promoted proliferation of tenocytes/Fig.2(E)WB/img868.jpg]

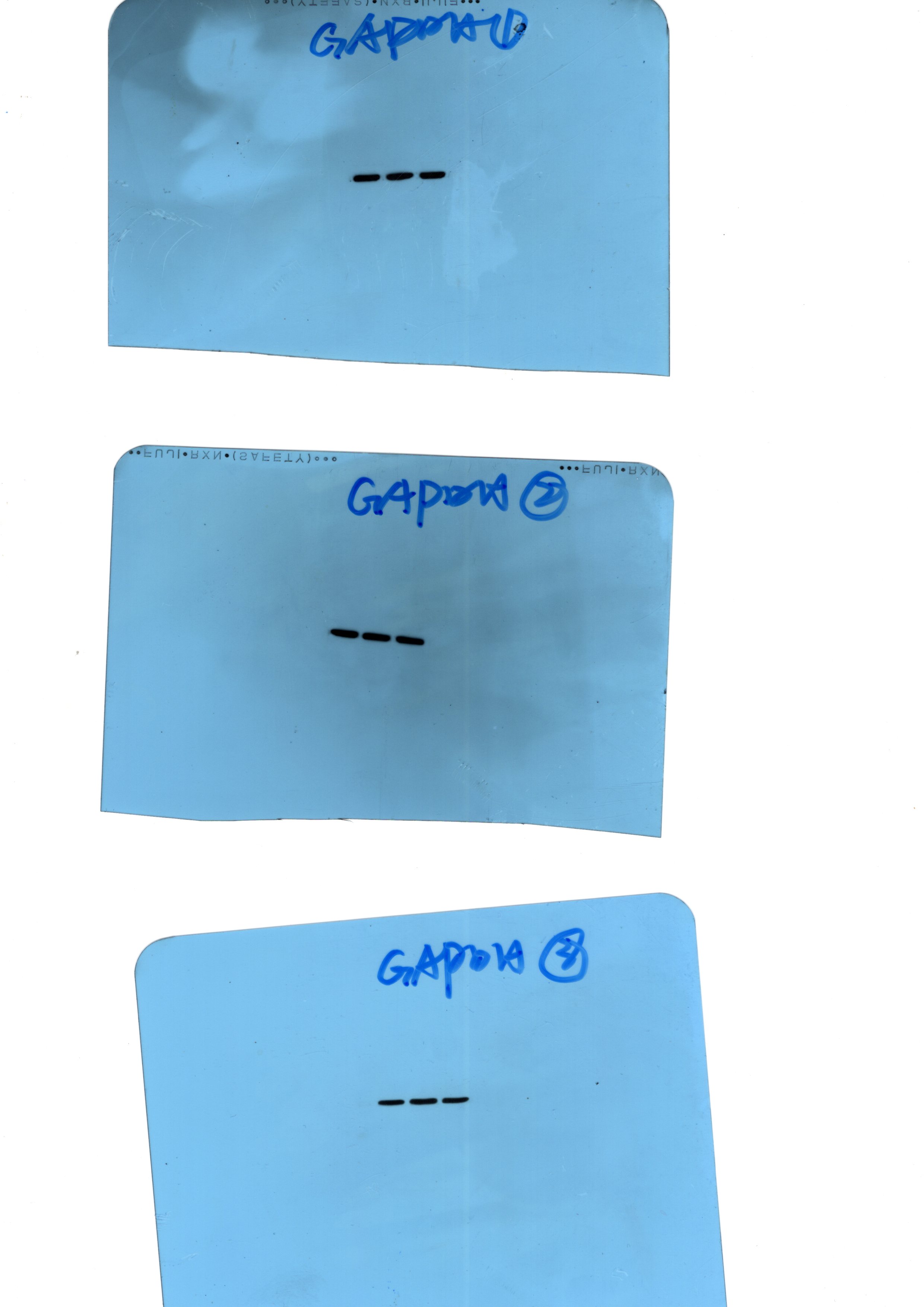

Supplement: Supplementary file 4 [file DataSheet2.ZIP › Fig. 2 LncRNA Morf4l1 inhibited oxidative stress and promoted proliferation of tenocytes/Fig.2(E)WB/img869.jpg]

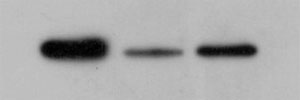

Supplement: Supplementary file 4 [file DataSheet2.ZIP › Fig. 2 LncRNA Morf4l1 inhibited oxidative stress and promoted proliferation of tenocytes/Fig.2(E)WB/PCNA(1).jpg]

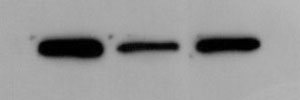

Supplement: Supplementary file 4 [file DataSheet2.ZIP › Fig. 2 LncRNA Morf4l1 inhibited oxidative stress and promoted proliferation of tenocytes/Fig.2(E)WB/PCNA(2).jpg]

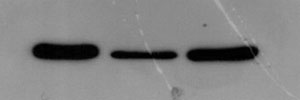

Supplement: Supplementary file 4 [file DataSheet2.ZIP › Fig. 2 LncRNA Morf4l1 inhibited oxidative stress and promoted proliferation of tenocytes/Fig.2(E)WB/PCNA(3).jpg]

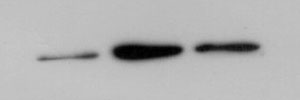

Supplement: Supplementary file 5 [file DataSheet5.ZIP › Fig. 5 ADSCs promoted tendon wound healing in vivo/Fig.5(C)WB/Bax(1).jpg]

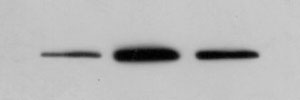

Supplement: Supplementary file 5 [file DataSheet5.ZIP › Fig. 5 ADSCs promoted tendon wound healing in vivo/Fig.5(C)WB/Bax(2).jpg]

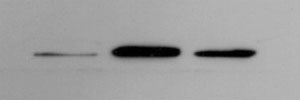

Supplement: Supplementary file 5 [file DataSheet5.ZIP › Fig. 5 ADSCs promoted tendon wound healing in vivo/Fig.5(C)WB/Bax(3).jpg]

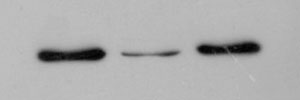

Supplement: Supplementary file 5 [file DataSheet5.ZIP › Fig. 5 ADSCs promoted tendon wound healing in vivo/Fig.5(C)WB/BCL-2(1).jpg]

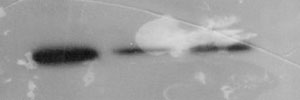

Supplement: Supplementary file 5 [file DataSheet5.ZIP › Fig. 5 ADSCs promoted tendon wound healing in vivo/Fig.5(C)WB/BCL-2(2).jpg]

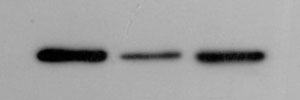

Supplement: Supplementary file 5 [file DataSheet5.ZIP › Fig. 5 ADSCs promoted tendon wound healing in vivo/Fig.5(C)WB/BCL-2(3).jpg]

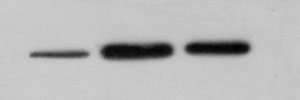

Supplement: Supplementary file 5 [file DataSheet5.ZIP › Fig. 5 ADSCs promoted tendon wound healing in vivo/Fig.5(C)WB/Caspase3(1).jpg]

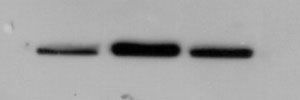

Supplement: Supplementary file 5 [file DataSheet5.ZIP › Fig. 5 ADSCs promoted tendon wound healing in vivo/Fig.5(C)WB/Caspase3(2).jpg]

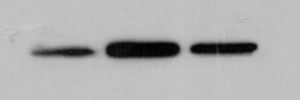

Supplement: Supplementary file 5 [file DataSheet5.ZIP › Fig. 5 ADSCs promoted tendon wound healing in vivo/Fig.5(C)WB/Caspase3(3).jpg]

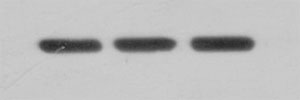

Supplement: Supplementary file 5 [file DataSheet5.ZIP › Fig. 5 ADSCs promoted tendon wound healing in vivo/Fig.5(C)WB/GAPDH(1).jpg]

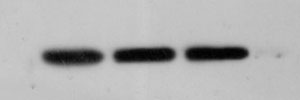

Supplement: Supplementary file 5 [file DataSheet5.ZIP › Fig. 5 ADSCs promoted tendon wound healing in vivo/Fig.5(C)WB/GAPDH(2).jpg]

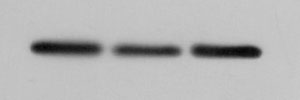

Supplement: Supplementary file 5 [file DataSheet5.ZIP › Fig. 5 ADSCs promoted tendon wound healing in vivo/Fig.5(C)WB/GAPDH(3).jpg]

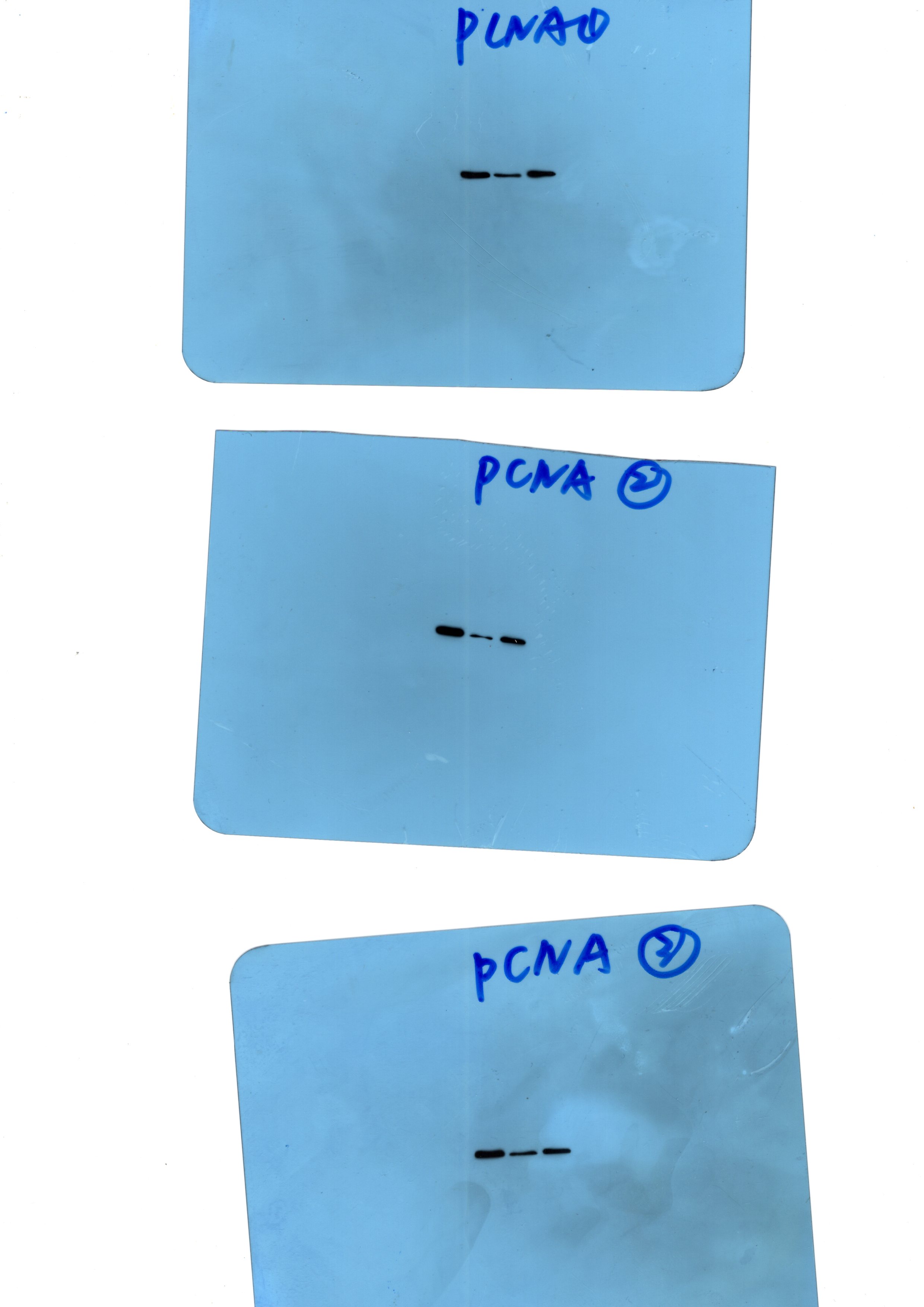

Supplement: Supplementary file 5 [file DataSheet5.ZIP › Fig. 5 ADSCs promoted tendon wound healing in vivo/Fig.5(C)WB/img879.jpg]

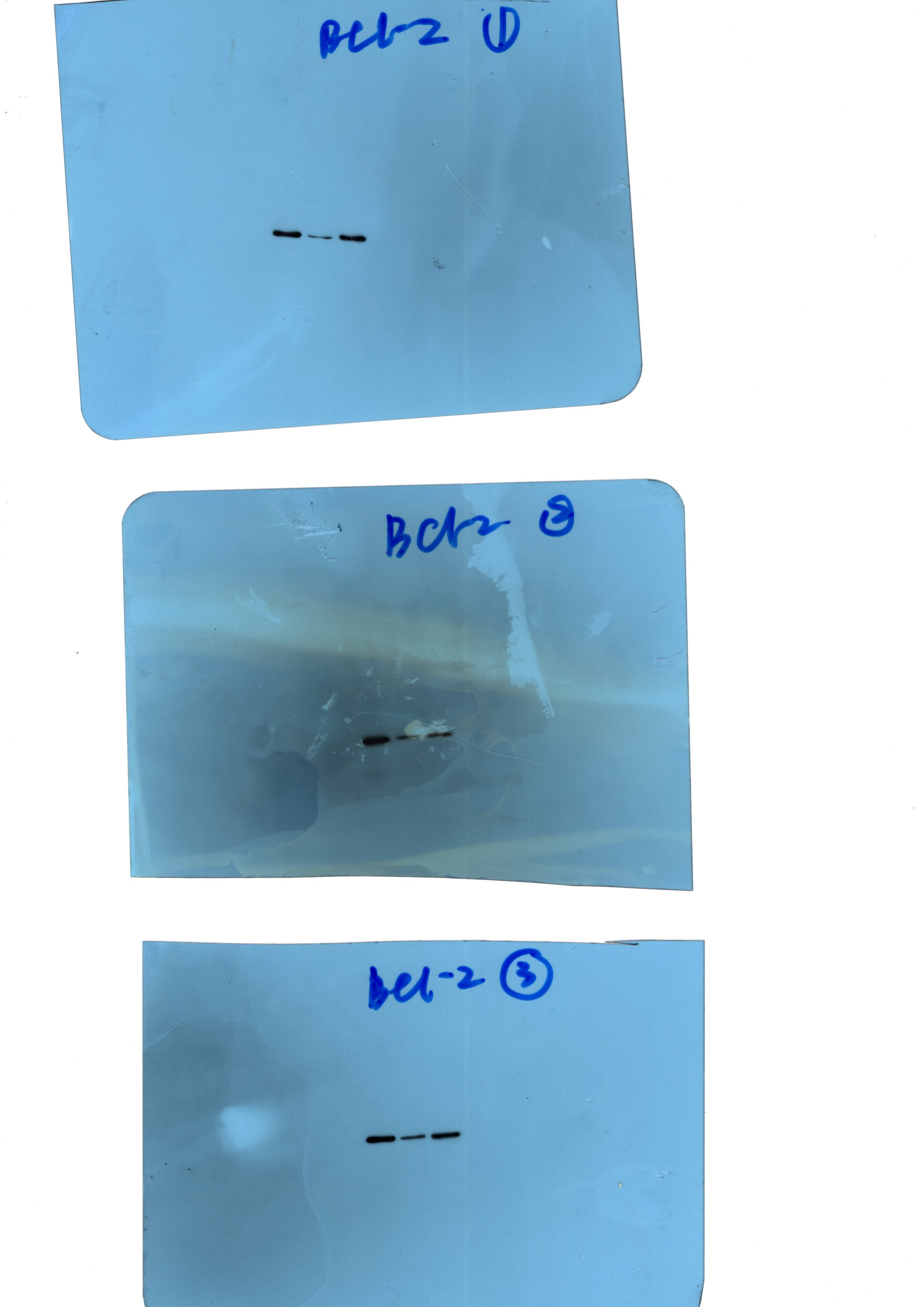

Supplement: Supplementary file 5 [file DataSheet5.ZIP › Fig. 5 ADSCs promoted tendon wound healing in vivo/Fig.5(C)WB/img880.jpg]

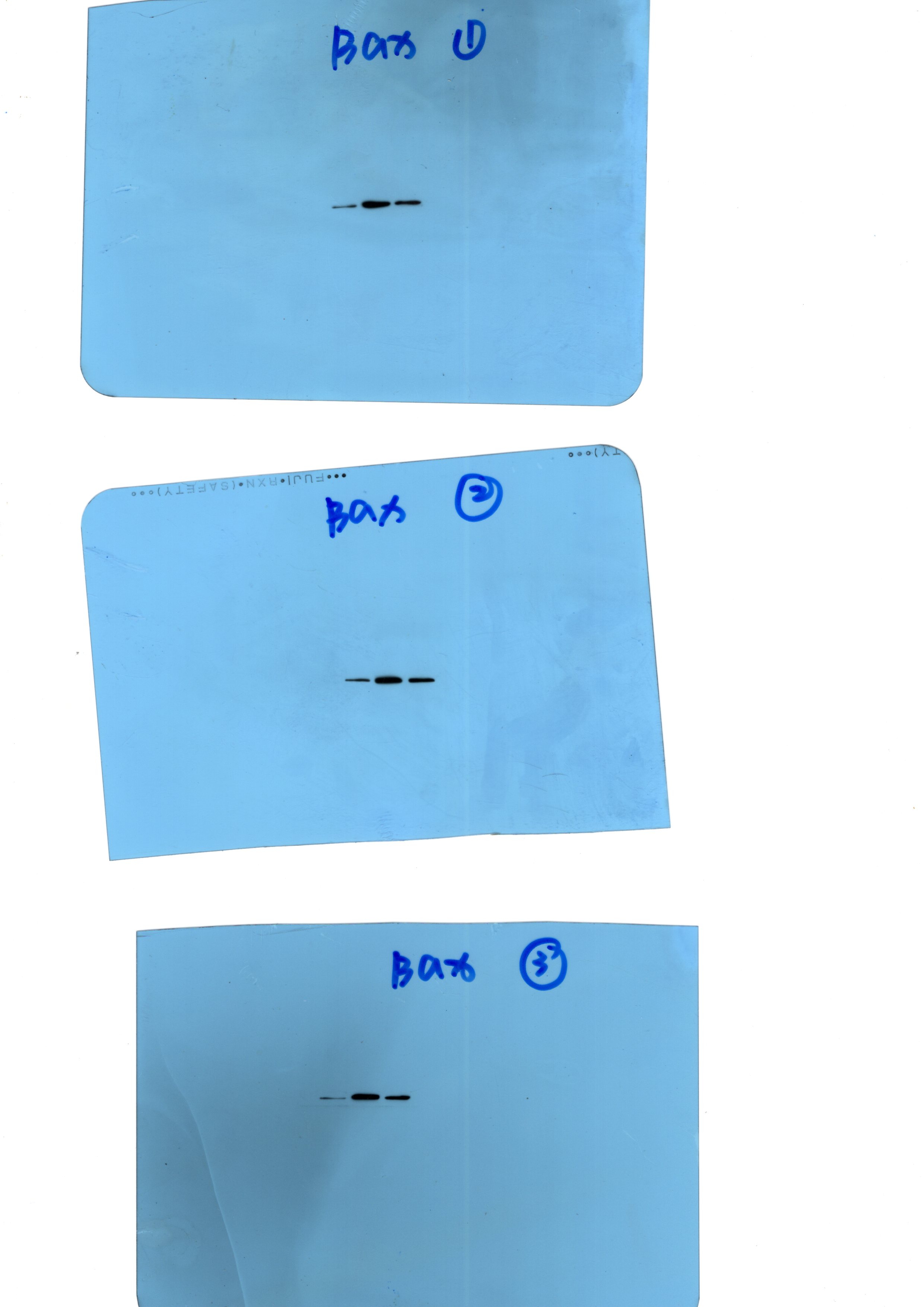

Supplement: Supplementary file 5 [file DataSheet5.ZIP › Fig. 5 ADSCs promoted tendon wound healing in vivo/Fig.5(C)WB/img881.jpg]

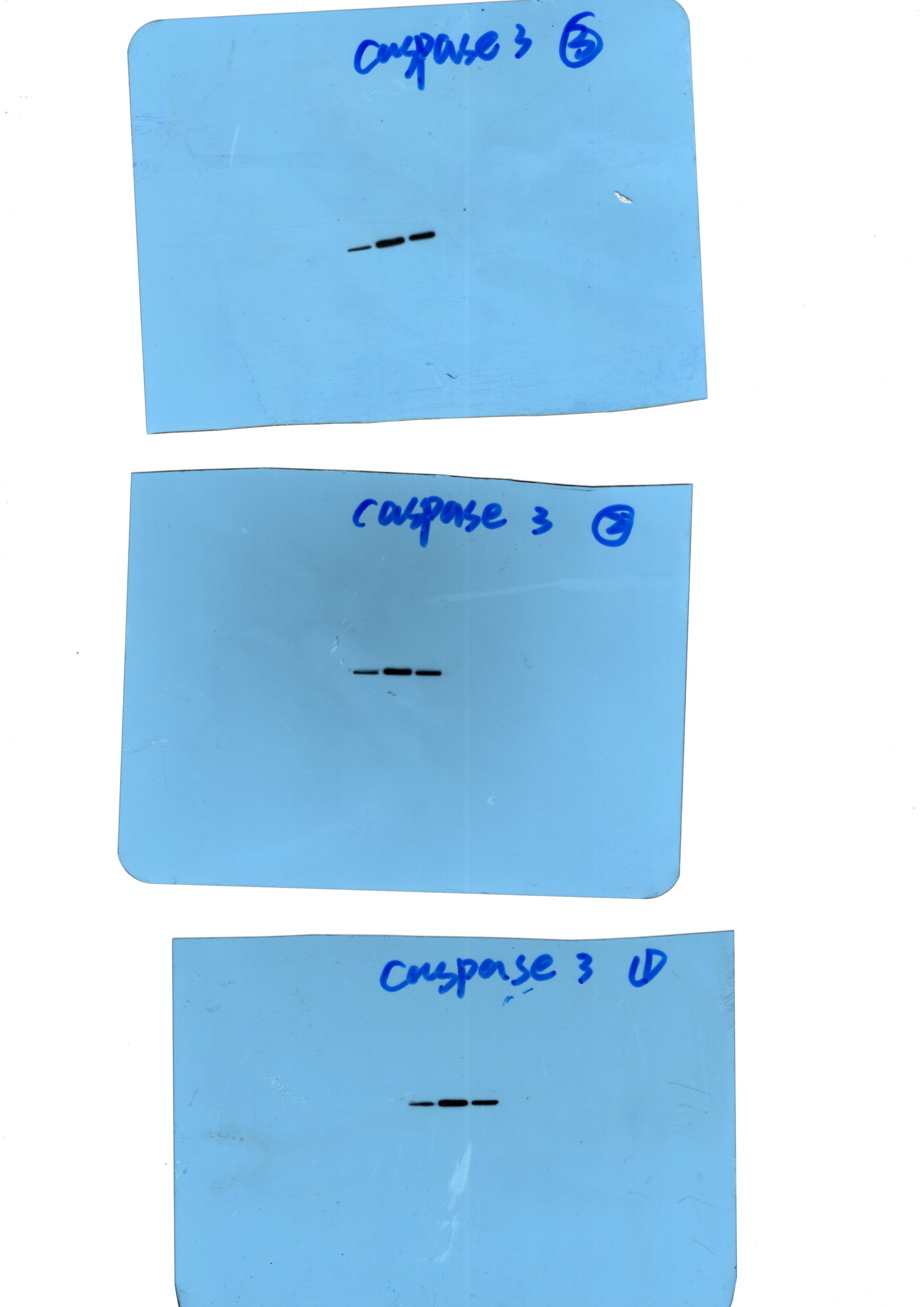

Supplement: Supplementary file 5 [file DataSheet5.ZIP › Fig. 5 ADSCs promoted tendon wound healing in vivo/Fig.5(C)WB/img882.jpg]

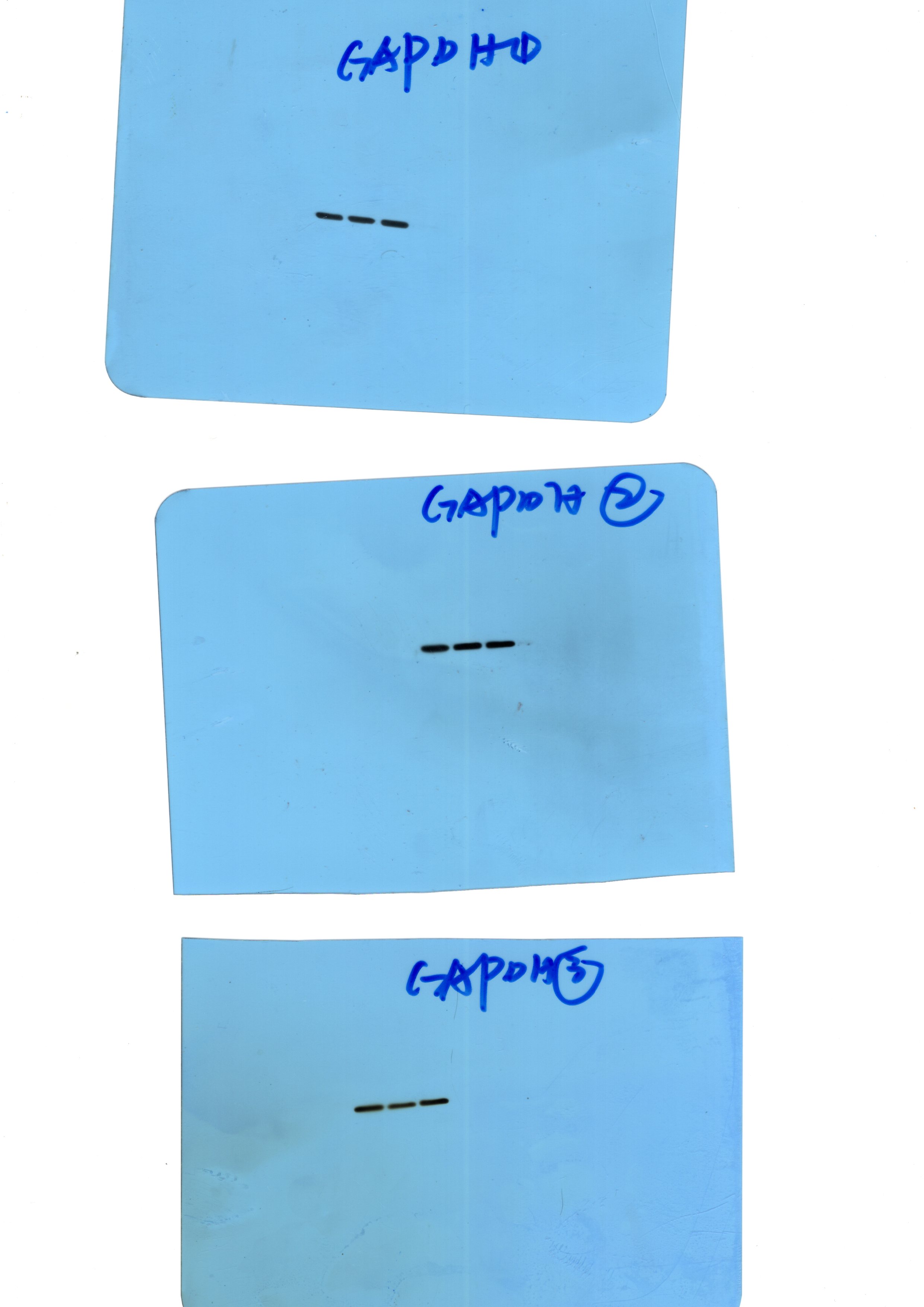

Supplement: Supplementary file 5 [file DataSheet5.ZIP › Fig. 5 ADSCs promoted tendon wound healing in vivo/Fig.5(C)WB/img883.jpg]

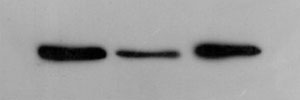

Supplement: Supplementary file 5 [file DataSheet5.ZIP › Fig. 5 ADSCs promoted tendon wound healing in vivo/Fig.5(C)WB/PCNA(1).jpg]

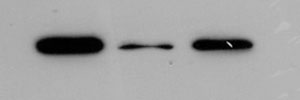

Supplement: Supplementary file 5 [file DataSheet5.ZIP › Fig. 5 ADSCs promoted tendon wound healing in vivo/Fig.5(C)WB/PCNA(2).jpg]

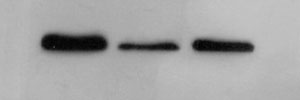

Supplement: Supplementary file 5 [file DataSheet5.ZIP › Fig. 5 ADSCs promoted tendon wound healing in vivo/Fig.5(C)WB/PCNA(3).jpg]
